# The Colibactin Genotoxin Generates DNA Interstrand Cross-Links in Infected Cells

**DOI:** 10.1128/mBio.02393-17

**Published:** 2018-03-20

**Authors:** Nadège Bossuet-Greif, Julien Vignard, Frédéric Taieb, Gladys Mirey, Damien Dubois, Claude Petit, Eric Oswald, Jean-Philippe Nougayrède

**Affiliations:** a IRSD, Université de Toulouse, INSERM, INRA, ENVT, UPS, Toulouse, France; b Toxalim, INRA, Toulouse, France; Yale University School of Medicine; Pasteur Institute

**Keywords:** DNA cross-linking agents, DNA damage, DNA damage checkpoints, *Escherichia coli*, *Escherichia* toxins, genotoxicity

## Abstract

Colibactins are hybrid polyketide-nonribosomal peptides produced by *Escherichia coli*, *Klebsiella pneumoniae*, and other *Enterobacteriaceae* harboring the *pks* genomic island. These genotoxic metabolites are produced by *pks*-encoded peptide-polyketide synthases as inactive prodrugs called precolibactins, which are then converted to colibactins by deacylation for DNA-damaging effects. Colibactins are bona fide virulence factors and are suspected of promoting colorectal carcinogenesis when produced by intestinal *E. coli*. Natural active colibactins have not been isolated, and how they induce DNA damage in the eukaryotic host cell is poorly characterized. Here, we show that DNA strands are cross-linked covalently when exposed to enterobacteria producing colibactins. DNA cross-linking is abrogated in a *clbP* mutant unable to deacetylate precolibactins or by adding the colibactin self-resistance protein ClbS, confirming the involvement of the mature forms of colibactins. A similar DNA-damaging mechanism is observed *in cellulo*, where interstrand cross-links are detected in the genomic DNA of cultured human cells exposed to colibactin-producing bacteria. The intoxicated cells exhibit replication stress, activation of ataxia-telangiectasia and Rad3-related kinase (ATR), and recruitment of the DNA cross-link repair Fanconi anemia protein D2 (FANCD2) protein. In contrast, inhibition of ATR or knockdown of FANCD2 reduces the survival of cells exposed to colibactin-producing bacteria. These findings demonstrate that DNA interstrand cross-linking is the critical mechanism of colibactin-induced DNA damage in infected cells.

## IMPORTANCE

Colorectal cancer is the third-most-common cause of cancer death. In addition to known risk factors such as high-fat diets and alcohol consumption, genotoxic intestinal *Escherichia coli* bacteria producing colibactin are proposed to play a role in colon cancer development. Here, by using transient infections with genotoxic *E. coli*, we showed that colibactins directly generate DNA cross-links *in cellulo*. Such lesions are converted into double-strand breaks during the repair response. DNA cross-links, akin to those induced by metabolites of alcohol and high-fat diets and by widely used anticancer drugs, are both severely mutagenic and profoundly cytotoxic lesions. This finding of a direct induction of DNA cross-links by a bacterium should facilitate delineating the role of *E. coli* in colon cancer and engineering new anticancer agents.

## INTRODUCTION

Colibactins are genotoxic natural products of unknown structure produced by human commensal and extraintestinal pathogenic strains of *Enterobacteriaceae* and by other bacteria associated with different host organisms. Biosynthesis of colibactins is allowed by a highly conserved 54-kb genomic “*pks*” island found in *Escherichia coli*, *Klebsiella pneumoniae*, *Enterobacter aerogenes*, and *Citrobacter koseri* (1, 2). The colibactin synthesis pathway is also found in marine *Pseudovibrio* spp. associated with sponges, in the honey bee gut symbiont *Frischella perrara*, and in *Erwinia oleae* isolated from olive tree knots (3–5). Colibactin production has been linked to pathogenicity and cancer. Indeed, the *pks* island is associated with a highly virulent subset of extraintestinal pathogenic *E. coli* isolates and with hypervirulent *K. pneumoniae* strains (6–8). The contribution of colibactins to the virulence of *E. coli* and *K. pneumoniae* has been demonstrated in rodent sepsis and meningitis models, where isogenic mutants impaired for colibactin production exhibit attenuated virulence compared to wild-type *pks*-positive (*pks*^*+*^) strains (9–11). The presence of the *pks* island also correlates with the ability of *E. coli* strains belonging to phylogenetic group B2 to establish persistent colonization of the host intestine (12). Studies have found an increased presence of *pks*^*+*^ *E. coli* in colorectal cancer patients (13–15). Furthermore, colonization with colibactin-producing *pks*^*+*^ *E. coli* promotes colon tumor formation in mouse models of chronic intestinal inflammation, supporting the idea of a role for these bacteria in the development of colorectal cancer (13, 16, 17).

The *pks* island hosts 19 genes (*clbA* to *clbS*), and mutation of any of these genes, except *clbS*, results in a decrease in or loss of the genotoxic activity (1). The *pks* island codes for multienzymatic biosynthesis machinery, with nonribosomal peptide synthetases (NRPSs) and polyketide synthases (PKSs), an efflux pump, and “accessory” enzymes. The NRPSs and PKSs are modified posttranslationally for acceptance of synthesis building blocks by the ClbA phosphopantetheinyl transferase (18, 19). The NRPSs and PKSs then function as a multimodular assembly line that synthesizes the inactive precolibactins. Precolibactin biosynthetic intermediates are offloaded from the assembly line by the ClbQ thioesterase, thus possibly regulating colibactin synthesis and genotoxic activity (20, 21). Precolibactins then follow a prodrug pathway, where the products are transported to the bacterial periplasm by the ClbM pump (22). Precolibactins are cleaved by the periplasmic peptidase ClbP to remove an N-terminal precursor scaffold (myristoyl-asparagine) and generate active colibactins (23–25). This prodrug export and activation pathway likely represents a self-protection mechanism preventing self-toxicity to the producing bacteria. As a supplemental self-protection mechanism, the *pks* island encodes the ClbS resistance protein, a cyclopropane hydrolase that inactivates colibactins in the producing bacterium (26, 27). This protection is also observed in cultured human cells transfected with the *clbS* gene (26).

Potent colibactin genotoxicity has been observed in mammalian cells transiently infected with *pks*^+^ enterobacteria: a 4-h contact between live *pks*^+^ *E. coli* and cultured epithelial cells results in host DNA double-strand breaks (DSBs) that can be detected 4 h later (1). As a result, the cell recruits the DNA-damage response and activates the ataxia-telangiectasia mutated kinase (ATM) pathway, resulting in cell cycle arrest to allow DNA repair or in removal of the injured cells by apoptosis and senescence (1, 16, 28, 29). *In vivo*, signs of DNA damage can be detected in gut cells of animals colonized with *pks*^+^ *E. coli* (10, 28, 30) or in liver or brain cells of mice infected with *pks*^+^ *K. pneumoniae* (11, 31). The DNA damage inflicted by colibactins is deleterious for the host cell genome stability, as low numbers of infecting *pks*^+^ *E. coli* bacteria promote chromosomal instability, aneuploidy, and gene mutations, resulting in cellular transformation (28). As natural mature colibactins confound isolation, how they inflict DNA damage, directly or indirectly, leading to the formation of DNA DSBs and ultimately promoting tumorigenesis, remains poorly understood. In this study, we identified DNA interstrand cross-links (ICLs) as the primary mechanism by which the naturally bacterially produced colibactins induce DNA damage in purified DNA *in vitro* and in infected cultured human cells *in cellulo*.

## RESULTS

### Extracellular DNA protects HeLa cells from the genotoxicity of *pks*^+^ *E. coli* and exhibits interstrand cross-links.

**FIG 1:**
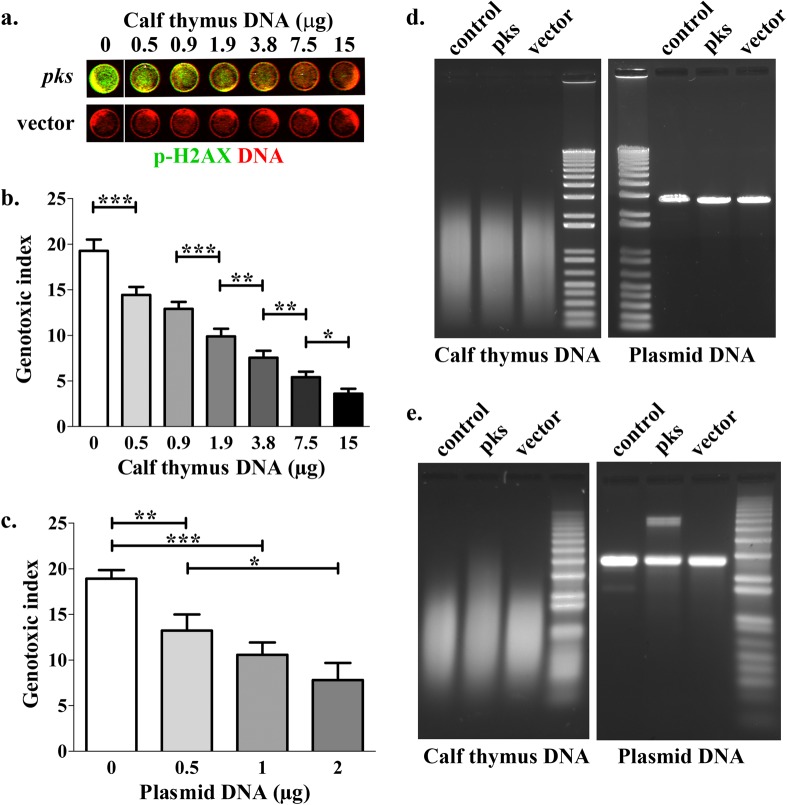
Exogenous DNA protects HeLa cells from the DNA damage induced by *pks*^+^ *E. coli* and displays nondenaturable interstrand cross-links. DH10B pBACpks (*pks*) or the vector was inoculated into HeLa cells (3 × 10^6^ bacteria in 100 μl, corresponding to a multiplicity of infection [MOI] of 200 bacteria/cell) in the presence of the indicated amounts of sonicated calf thymus DNA or linearized plasmid DNA. (a) After a 4-h infection followed by washes and 4 h of incubation with gentamicin, HeLa cell DNA double-strand breaks were revealed by staining p-H2AX. (b and c) Genotoxic index values were calculated for calf thymus DNA (b) and plasmid DNA (c) as phosphorylated H2AX signal relative to DNA content, normalized to control cells. Means and standard deviations of results from four independent experiments are shown. *, *P* < 0.05; **, *P* < 0.01; ***, *P* < 0.001 (one-way ANOVA and Bonferroni posttest). (d and e) The supernatants of cells infected for 4 h (MOI = 200) in the presence of 3.8 μg calf thymus DNA or 2 μg plasmid DNA were collected, centrifuged, and filtered to remove the bacteria, and the extracellular DNA was purified and analyzed by electrophoresis in 1% agarose gels under native (d) or alkaline denaturing (e) conditions.

It was recently proposed that an isolated candidate nongenotoxic precolibactin, or synthetic compounds analogous to predicted colibactins, could alkylate DNA (32, 33). We reasoned that if natural active colibactins produced by *pks*^+^ *E. coli* can alkylate and hence bind DNA, addition of extracellular DNA during the infection of mammalian cells should capture colibactins and thus inhibit their genotoxicity. To test this hypothesis, HeLa cells were infected for 4 h with laboratory *E. coli* strain DH10B hosting a bacterial artificial chromosome bearing the *pks* island (pBACpks), in the presence or absence of linear double-strand bacterial plasmid or calf thymus DNA in the culture medium. HeLa cell DNA damage was then quantified by examining the phosphorylation of histone H2AX on S139 (p-H2AX), a sensitive and quantitative reporter of DNA damage (34) (Fig. 1a). The addition of 500 ng of either calf thymus DNA or linearized plasmid DNA in the interaction medium readily reduced the p-H2AX levels in HeLa cells exposed to the genotoxic *pks*^+^ *E. coli*. Increasing the amount of extracellular DNA further decreased the p-H2AX response (Fig. 1b and c). These results suggest that the extracellular double-strand DNA captured the genotoxin, resulting in reduced DNA damage in HeLa cells exposed to colibactin-producing *E. coli*.

We next examined whether the extracellular DNA exposed to colibactin-producing bacteria exhibited damage. The plasmid or calf thymus DNA was isolated from the infection coculture medium and analyzed by native or denaturing agarose gel electrophoresis. The DNA did not show detectable cleaved DNA on nondenaturing gels (Fig. 1d). In contrast, following electrophoresis under alkaline denaturing conditions (thus allowing the migration of DNA in single-strand form), the DNA from the coculture composed of the *pks*^+^ *E. coli* and the HeLa cells exhibited DNA fragments with a marked decrease in their electrophoretic mobility (Fig. 1e). Calf thymus DNA fragments with an apparent molecular weight of more than 5 kb were detected in the DH10B pBACpks coculture, whereas DNA from the control uninfected culture or from the DH10B vector coculture remained at similar levels below 3.5 kb (Fig. 1e; see also Fig. S1 in the supplemental material). The plasmid DNA isolated from the *pks*^+^ *E. coli* HeLa cell coculture exhibited a supplementary band with a 2-fold apparent molecular weight increase (Fig. 1e; see also Fig. S1), suggesting that the DNA strands did not separate during electrophoresis despite the denaturing conditions. The DH10B vector strain did not induce this doubling in apparent molecular weight on the denaturing gels.

10.1128/mBio.02393-17.1 FIG S1  DNA signal intensities (gray values) of the electrophoretic gels shown in Fig. 1. The images were analyzed using NIH ImageJ. The background was subtracted with a rolling-ball radius of 100 pixels (px), each lane was selected with a pasted rectangular selection, and the corresponding plot profile (shortcut ALT-K) was determined. The migration distances were calibrated in apparent molecular weight values, based on the molecular weight marker profile, using VisualSpec software. On the lower left panel, the highest molecular weight (bases) of the DNA signal detected above 5 times that of the average background signal is shown. On the lower right panel, the apparent molecular weights of the bands, as determined by the center of the Gaussian fit, are shown. Download FIG S1, TIF file, 0.7 MB. Copyright © 2018 Bossuet-Greif et al. 2018 Bossuet-Greif et al. This content is distributed under the terms of the Creative Commons Attribution 4.0 International license.

**FIG 2:**
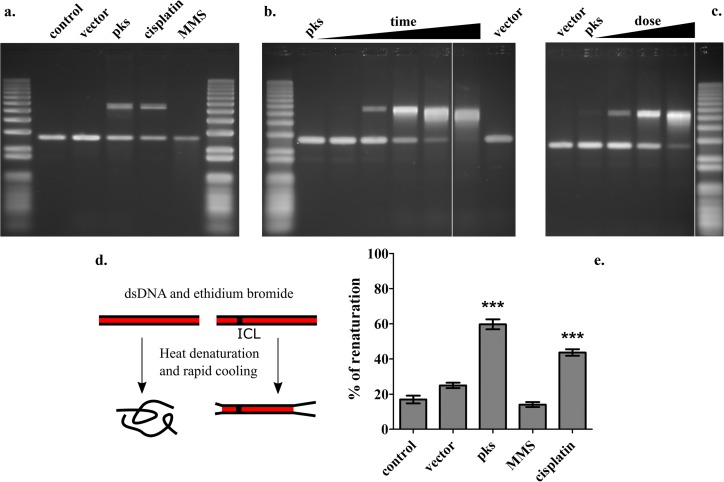
Nondenaturable interstrand cross-links in DNA exposed to *pks*^+^ *E. coli*. (a) Linearized plasmid double-strand DNA (400 ng) was incubated for 4 h with DH10B pBAC*pks* or vector (inoculum of 3 × 10^6^ bacteria in 100 μl) or treated for 4 h with 80 μM cisplatin or 5 mM methyl methanesulfonate (MMS) and then analyzed by denaturing gel electrophoresis. (b) The DNA was exposed for 1, 2, 3, 4, 5, or 6 h to DH10B pBACpks (inoculum of 3 × 10^6^ bacteria in 100 μl) or for 6 h to DH10B vector. (c) The DNA was exposed for 4 h to various numbers of *E. coli* DH10B pBACpks (inocula of 0.75, 1.5, 3, and 6 × 10^6^ bacteria in 100 μl) or vector (6 × 10^6^ bacteria). The molecular size marker in panel c and a duplicate lane between lanes 7 and 8 in panel b were moved and removed, respectively, during figure assembly. (d) Fluorescence assay to detect covalent DNA interstrand cross-links. The double-stranded DNA (dsDNA) was stained with ethidium bromide, subjected to heat denaturation at 95°C, and then cooled to 23°C in less than 1 min, conditions under which separable DNA strands do not reanneal. The percentage of DNA renaturation was calculated as the ratio of the level of ethidium fluorescence determined after heating to the level of the fluorescence before heating. (e) The percentage of renaturation was measured in DNA treated as described for panel a. Means and standard deviations of results from five independent experiments are shown. ***, *P* < 0.01 (compared to control; one-way ANOVA and Bonferroni posttest).

Plasmid DNA exposed for 4 h to live *pks*^+^ *E. coli* without the eukaryotic cells also exhibited DNA fragments that had shifted to a 2-fold apparent molecular weight increase on denaturing gels (Fig. 2a). When the DNA was exposed to *pks*^+^ *E. coli* with increased incubation times or with increased numbers of bacteria, the proportion of the shifted band increased while that of the unmodified lower DNA band diminished (Fig. 2b and c). In contrast, the DNA exposed to control *E. coli* hosting the bacterial artificial chromosome (BAC) vector under identical conditions did not show such an electrophoretic mobility shift (Fig. 2b and c). The DNA shift induced by exposure to *pks*^+^ *E. coli* was similar to that induced by treatment with the DNA cross-linking drug cisplatin, whereas the monofunctional alkylating agent methyl methanesulfonate (MMS) did not induce a detectable band shift (Fig. 2a). These results strongly suggest that exposure to *pks*^+^ *E. coli* bacteria generated DNA interstrand cross-links (ICLs).

To confirm the occurrence of ICLs in the extracellular DNA exposed to *pks*^+^ *E. coli*, we used an ethidium fluorescence assay to examine the recovery of fluorescence following denaturation and rapid cooling. In this assay, separate DNA strands do not reanneal, whereas ICL-connected strands renature rapidly, as ICLs serve as a nucleation points for rapid renaturation (35) (Fig. 2d). The DNA exposed to the *E. coli* vector or to MMS, which does not induce ICL, showed a modest renaturation similar to the results seen the control untreated DNA (Fig. 2e). In contrast, DNA exposed to *pks*^+^ *E. coli* exhibited marked renaturation, similar to that in cisplatin-treated DNA (Fig. 2e). Taken together, the results from the alkaline electrophoresis and renaturation assay demonstrated that exposure of DNA to *pks*^+^ *E. coli* induces DNA ICL *in vitro*.

### **Colibactins produced by** ***pks***^+^ ***Enterobacteriaceae*** **generate interstrand cross-links in extracellular DNA.**

**FIG 3:**
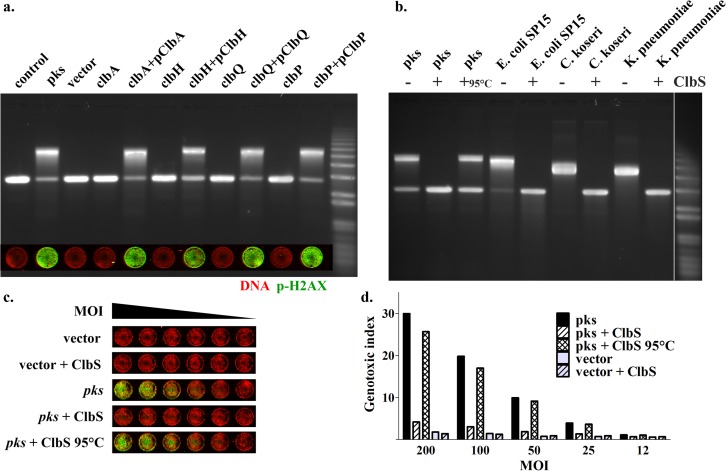
Colibactin synthesis pathway inactivation or addition of the purified colibactin self-resistance protein ClbS inhibits the *in vitro* DNA cross-linking and *in cellulo* DNA damage induced by *pks*^+^ enterobacteria. (a) Linearized double-stranded plasmid DNA was exposed for 4 h to DH10B pBAC*pks* or vector; the *clbA*, *clbH*, *clbQ*, and *clbP* isogenic mutants; or the mutants complemented with plasmids encoding the cognate wild-type genes (inoculum of 3 × 10^6^ bacteria in 100 μl). The DNA was analyzed by denaturing electrophoresis. (Inset) HeLa cells were infected for 4 h with each of the mutant and complemented strains (MOI = 200), washed, and incubated for 4 h with gentamicin, and then cellular DNA damage was revealed by staining of p-H2AX relative to nuclear DNA. (b) Linearized double-strand plasmid DNA was exposed to *E. coli* DH10B pBAC*pks* or vector or to clinical *E. coli* strain SP15, *Citrobacter koseri*, or *Klebsiella pneumoniae* (inoculum of 6 × 10^6^ bacteria in 100 µl) in the presence or absence of 400 nM purified 6-histidine-ClbS protein or the protein denatured by heating at 95°C. The DNA was collected and analyzed by denaturing electrophoresis. (c) HeLa cells were infected with DH10B pBAC*pks* or vector at an MOI of 12 to 200 in the presence of 400 nM of purified ClbS or the heat-denatured protein. After a 4-h infection followed by washes and 4 h of incubation with gentamicin, the cells were stained for p-H2AX and DNA. Genotoxic index values (p-H2AX signal relative to DNA content, normalized to control cells) are shown in panel d. The histogram bars represent means of results from a duplicate experiment.

We next sought to establish the role of colibactins in the formation of DNA ICL by *pks*^+^ *E. coli*. Direct contact of live *pks*^+^ *E. coli* cells producing colibactins with eukaryotic cells is required to observe nuclear DNA damage (1, 36). Similarly, the extracellular DNA cross-linking activity of *pks*^+^ *E. coli* required a close interaction with live bacteria; no ICLs were observed in the DNA treated with bacterial culture supernatants or in the DNA separated from the DH10B pBAC*pks* bacteria by a membrane with 0.2-μm pores (Fig. S2). The genotoxicity and interstrand cross-linking phenotypes were fully correlated in different pathogenic *Enterobacteriaceae* strains harboring the *pks* island; clinical strains of *E. coli*, *Citrobacter koseri*, *Klebsiella pneumoniae*, and *Enterobacter aerogenes* induced both p-H2AX in infected HeLa cells and ICL in extracellular DNA (Fig. S3). To further establish the role of colibactins in the induction of extracellular DNA ICL, we assessed isogenic mutants of *pks* island genes that impair various steps of the colibactin synthesis pathway. Mutation of the *clbA* gene encoding the phosphopantetheinyl transferase that activates the NRPS and PKS enzymes resulted in a complete loss of extracellular DNA interstrand cross-linking activity and genotoxicity in infected HeLa cells (Fig. 3a). A *clbH* mutant impaired for the NRPS that incorporates the electrophile cyclopropane in colibactin (32, 37) also exhibited a complete loss of both phenotypes (Fig. 3a). Mutation of *clbQ* encoding the thioesterase that off-loads synthesis intermediates from the NRPS-PKS enzymatic assembly line abrogated both phenotypes (Fig. 3a). Finally, a *clbP* mutant defective for the peptidase that deacetylates precolibactins to generate the mature active colibactins completely lost the cross-linking activity and genotoxicity for HeLa cells (Fig. 3a). Both the genotoxicity and cross-linking phenotypes were fully restored by complementation of all mutants with their cognate wild-type *clb* genes (Fig. 3a). Thus, an intact colibactin synthesis pathway is required for the DNA cross-linking activity.

10.1128/mBio.02393-17.2 FIG S2  DNA interstrand cross-links are generated in a contact-mediated manner by *pks*^+^ bacteria. Linearized double-stranded plasmid DNA was exposed to DH10B pBAC*pks* bacteria, to the bacterial culture supernatant, or to the live bacteria placed in an insertion separated from the DNA by a permeable membrane with 0.2-μm pores. Following exposure, the DNA was collected and analyzed by denaturing electrophoresis. Download FIG S2, TIF file, 0.5 MB. Copyright © 2018 Bossuet-Greif et al. 2018 Bossuet-Greif et al. This content is distributed under the terms of the Creative Commons Attribution 4.0 International license.

10.1128/mBio.02393-17.3 FIG S3  DNA interstrand cross-link formation by various *Enterobacteriaceae* species harboring the *pks* island. (a) *E. coli* DH10B pBAC*pks* or vector or clinical *Escherichia coli* strain SP15, *Citrobacter koseri* strain BAA-895, *Klebsiella pneumoniae* strain CF1, or *Enterobacter aerogenes* strain 64 was grown for 3.5 h (with inocula of 1.5 × 10^6^ bacteria in 100 µl for *E. coli* and 7.5 × 10^5^ bacteria in 100 μl for *C. koseri*, *K. pneumoniae*, and *E. aerogenes*), and then EDTA (1 mM final concentration) and 400 ng linearized double-strand plasmid DNA were added. Following a 40-min incubation at 37°C, the DNA was collected and analyzed on a denaturing 1% agarose gel. (b) HeLa cells were infected for 4 h with the corresponding enterobacteria (MOI = 200), washed, and incubated for 4 h with gentamicin, and then cellular DNA double-strand breaks were revealed by staining of S139-phosphorylated histone H2AX (green) relative to nuclear DNA (red). Download FIG S3, TIF file, 0.8 MB. Copyright © 2018 Bossuet-Greif et al. 2018 Bossuet-Greif et al. This content is distributed under the terms of the Creative Commons Attribution 4.0 International license.

To accumulate additional evidence to establish the role of colibactins in the induction of DNA ICL, we tested whether the colibactin resistance protein could protect DNA from the genotoxin. Indeed, colibactin-producing bacteria protect themselves from their own mature active genotoxin by producing the ClbS hydrolase, which inactivates colibactins (26, 27). We thus reasoned that adding the purified ClbS protein into the supernatant should inhibit colibactin-induced DNA ICL. DNA was exposed to *E. coli* DH10B pBAC*pks* or to the *E. coli* SP15, *C. koseri*, and *K. pneumoniae* clinical isolates in the presence or absence of 400 nM purified His-tagged ClbS protein. The DNA did not exhibit ICL (or the levels were barely detectable), indicating that it was readily protected by ClbS (Fig. 3b; see also Fig. S4). In contrast, the heat-denatured ClbS protein did not protect the DNA exposed to the *pks*^+^ bacteria (Fig. 3b; see also Fig. S4). ClbS did not inhibit DNA cross-linking by cisplatin, confirming its specificity for colibactins (Fig. S4). HeLa cells infected with DH10B pBACpks in the presence of purified ClbS exhibited markedly reduced levels of p-H2AX compared to cells infected in the presence of heat-denatured ClbS or without ClbS (Fig. 3c and d), confirming that ClbS inhibits the genotoxin activity. Taken together, the results from these genetic analyses and protection assays show that the natural mature colibactins produced by *pks*^+^ *Enterobacteriaceae* generate ICLs in extracellular DNA.

10.1128/mBio.02393-17.4 FIG S4  The purified colibactin self-resistance protein ClbS inhibits the DNA cross-linking by *pks*^+^ *E. coli* but not by cisplatin. Linearized double-strand plasmid DNA was exposed to *E. coli* DH10B pBAC*pks* or vector (inoculum of 6 × 10^6^ bacteria in 100 µl) or was treated with 80 μM cisplatin in the presence or absence of 400 nM of purified 6-histidine-ClbS protein or of the protein denatured by heating at 95°C. The DNA was collected and analyzed by denaturing electrophoresis. The DNA signal in the upper, cross-linked band, relative to the total DNA signal in the lane, was determined by image analysis in 3 to 4 independent experiments. *, *P* < 0.05; **, *P* < 0.01 (one-way ANOVA and Bonferroni posttest). Download FIG S4, TIF file, 0.4 MB. Copyright © 2018 Bossuet-Greif et al. 2018 Bossuet-Greif et al. This content is distributed under the terms of the Creative Commons Attribution 4.0 International license.

### Infection of HeLa cells with colibactin-producing *E. coli* induces an ATR-dependent replication stress response.

**FIG 4:**
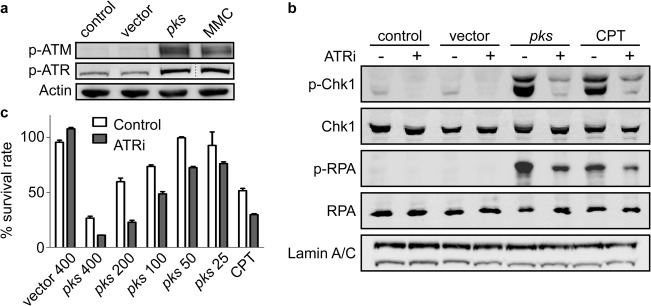
ATR signaling response in HeLa cells infected with *pks*^+^ *E. coli*. (a) HeLa cells were exposed for 4 h to *E. coli* DH10B hosting BACpks or vector (MOI = 200) or treated with MMC. Eight hours after infection, the activation of the DNA damage pathway was examined by Western blotting with the indicated antibodies. (b) HeLa cells were infected as described above or treated with cisplatin (CPT) and then treated or not with an ATR inhibitor (ATRi) before analysis. (c) The cell survival rate 48 h following infection and treatment with ATRi was assessed by staining the cells and quantifying the dye. Means and standard deviations of results from a triplicate experiment are shown.

We next asked whether colibactins could induce DNA ICL in human cells. We previously showed that colibactin-dependent genomic injuries induce the host DNA damage response through increases of levels of p-H2AX and activation of ATM by phosphorylation on S1981 (1). ATM activation depends on the presence of DSBs, which are DNA lesions that can be generated during replication-dependent ICL repair (38). Before DSB formation, ICLs first block progression of replication forks (by preventing replicative helicase-dependent double-strand DNA unwinding), resulting in the activation of ataxia-telangiectasia and Rad3-related kinase (ATR) by autophosphorylation at T1989. Activated ATR then coordinates the cellular response to the replication stress and ICL removal (39). We thus monitored ATR and ATM activation in HeLa cells collected 8 h after infection with DH10B hosting the BAC*pks* or the vector or after treatment with mitomycin C (MMC). Western blot analyses showed that both ATM and ATR were phosphorylated following infection with *pks*^+^ *E. coli* or after MMC treatment (Fig. 4a; see also Fig. S5). This indicated that, similarly to the results seen with MMC, infection with colibactin-producing bacteria induces the formation of replication stress-mediated DSBs.

10.1128/mBio.02393-17.5 FIG S5  Quantification of the results of the Western blot experiments presented in Fig. 4. The relative densities of the bands for p-ATM, p-ATR, p-Chk1, and p-RPA relative to actin or Chk1 or RPA were measured using NIH ImageJ. Each point corresponds to results of an independent experiment. Download FIG S5, TIF file, 0.1 MB. Copyright © 2018 Bossuet-Greif et al. 2018 Bossuet-Greif et al. This content is distributed under the terms of the Creative Commons Attribution 4.0 International license.

To characterize the ATR-dependent signaling response induced by *pks*^+^ *E. coli*, we next examined two targets of the ATR kinase activity, Chk1 and replication protein A-32 (RPA), for phosphorylation at S345 and S33, respectively (40, 41). As seen with cisplatin, but in contrast to the results seen with bacteria hosting the vector, infection with DH10B pBAC*pks* induced the phosphorylation of Chk1 and RPA. Inactivating ATR by the use of its specific inhibitor VE-821 (ATRi) (41) prevented this response (Fig. 4b; see also Fig. S5). We observed a loss of histone H3 phosphorylation (a hallmark of chromosome condensation) and of mitotic cells (Fig. S6) following infection with DH10B pBAC*pks* that was consistent with cell cycle arrest (1). Treatment with ATRi alleviated this response, allowing *pks*^+^ *E. coli*-infected cells to enter mitosis with damaged DNA, evidenced by p-H2AX staining (Fig. S6). These mitotic cells never reached anaphase, strongly suggesting the presence of a chromosome segregation defect that ultimately leads to cell death by mitotic catastrophe (42). Hence, ATRi impaired cell viability upon treatment with cisplatin or infection with *pks*^+^ bacteria but not infection with bacteria lacking *pks* (Fig. 4c). Together, these results showed that infection of human cells with colibactin-producing bacteria elicits an ATR-dependent replication stress response, similar to that promoted by ICL-inducing agents, resulting in cell cycle arrest to prevent mitosis entry with damaged chromosomes and, ultimately, mitotic catastrophe.

10.1128/mBio.02393-17.6 FIG S6  Inhibition of ATR allows mitosis reentry and catastrophe in HeLa cells infected with *pks*^+^ *E. coli*. (a) HeLa cells were exposed for 4 h to *E. coli* DH10B hosting the BACpks or vector (MOI = 200) and then treated or not with an ATR inhibitor (ATRi). At 8 h after infection, mitosis and DNA damage were examined by Western blotting with the indicated antibodies. (b) Representative images of DNA (DAPI staining) of HeLa cells infected for 4 h (MOI = 20) and then washed, treated or not with ATRi, and incubated for 20 h. Scale bar = 20 μm. Results of p-H2AX immunostaining (green) and DNA DAPI staining (red) of mitotic HeLa cells treated with ATRi are shown in the lower panel. Download FIG S6, TIF file, 1.6 MB. Copyright © 2018 Bossuet-Greif et al. 2018 Bossuet-Greif et al. This content is distributed under the terms of the Creative Commons Attribution 4.0 International license.

### HeLa cells infected with colibactin-producing *E. coli* exhibit recruitment of the Fanconi anemia repair pathway.

**FIG 5:**
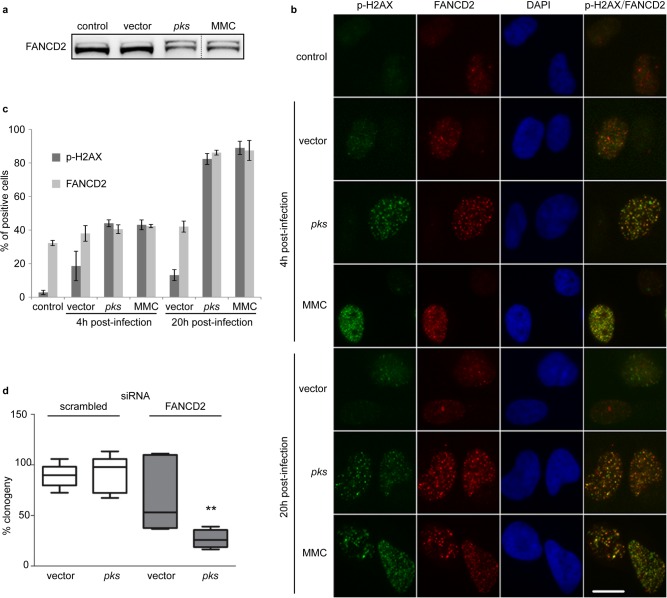
Recruitment of the cross-link repair protein FANCD2 in HeLa cells infected with *pks*^+^ *E. coli*. (a) HeLa cells were exposed for 4 h to *E. coli* DH10B hosting BACpks or vector (MOI = 200) or treated with mitomycin C (MMC). At 8 h after infection, the modification of FANCD2 was examined by Western blotting. The upper FANCD2 band was formed by monoubiquitination. A duplicate lane between lanes 3 and 4 was removed during figure assembly. (b) Representative images of p-H2AX and FANCD2 immunostaining in HeLa cells infected for 4 h (MOI = 20) or treated with 2.5 μM MMC and then washed and incubated for 4 or 20 h. DNA was counterstained with DAPI. Scale bar = 20 μm. (c) Cells positive for FANCD2 or p-H2AX were counted; means and standard deviations of results from at least 3 independent experiments are shown. (d) HeLa cells were transfected with scrambled or FANCD2 siRNAs and infected for 4 h (MOI = 12), 300 cells/well were seeded in 6-well plates and grown for 7 days, and colonies formed by surviving cells were counted. The box-and-whisker plots represent the percentages (median, interquartile, and minimum and maximum [min/max] values) of clonogenic cell survival relative to control cells in three independent experiments. **, *P* < 0.01 (one-way ANOVA with Bonferroni posttest).

In mammalian cells, the Fanconi anemia pathway is central to the repair of DNA ICL. Upon blockage of replication fork progression by DNA ICLs, Fanconi anemia protein D2 (FANCD2) is activated by monoubiquitinylation and is recruited to stalled forks, where it colocalizes in subnuclear foci with p-H2AX and orchestrates ICL removal (43–46). We thus examined whether HeLa cells infected with *pks*^+^ *E. coli* recapitulated these responses. As visualized by Western blotting, DH10B pBAC*pks*-infected and MMC-treated cells exhibited the heaviest form of FANCD2 corresponding to the monoubiquitinylated protein, whereas DH10B pBAC vector-infected cells exhibited mostly the unmodified form of FANCD2 (Fig. 5a). Next, FANCD2 and p-H2AX focus formation was examined by fluorescence microscopy in HeLa cells infected with DH10B pBAC*pks* or vector or treated with MMC. Four hours after exposure to DH10B vector, the cells presented some detectable p-H2AX and FANCD2 foci, reflecting the S-phase cells, as some FANCD2 foci form spontaneously during unperturbed replication (45) (Fig. 5b). In contrast, cells infected with DH10B pBAC*pks* exhibited distinctive nuclear foci of FANCD2 colocalized with p-H2AX, similarly to the results observed in MMC-treated cells (Fig. 5b; see also Fig. S7). The percentage of cells harboring p-H2AX and FANCD2 foci increased markedly between 4 and 20 h after infection with DH10B pBAC*pks* or treatment with MMC (Fig. 5c; see also Fig. S7). These comparable time-dependent responses strongly suggest that, similarly to the results seen with MMC-induced replication stress, DH10B pBAC*pks*-infected cells entering the S phase encountered ICLs that activated the Fanconi pathway. We confirmed these observations by assessing the recruitment to DH10B pBAC*pks*-induced repair foci of p53-binding protein 1 (53BP1) and RPA, two well-established markers of DNA DSBs and blocked replication forks, respectively. In the same way as was seen with p-H2AX/FANCD2 foci, 53BP1 and RPA colocalized in subnuclear foci in a time-dependent manner in MMC-treated or *pks*^+^ *E. coli*-infected cells but not after infection with *E. coli* hosting the pBAC vector (Fig. S8). Next, we verified that activation of the Fanconi anemia pathway upon infection with DH10B pBAC*pks* was mediated by an ATR-dependent replication stress response (46). Addition of ATRi to cisplatin-treated or *pks*^+^ *E. coli*-infected cells abrogated FANCD2 monoubiquitinylation and focus formation (Fig. S9). Thus, colibactins induce the replication stress response and recruitment of the Fanconi anemia pathway.

10.1128/mBio.02393-17.7 FIG S7  Quantification of the doubly positive FANCD2 and p-H2AX foci (shown in Fig. 5b) at 4 and 20 h after infection with DH10B vector or pBACpks (MOI = 20) or after treatment with 2.5 μM mitomycin c (MMC). Download FIG S7, TIF file, 0.2 MB. Copyright © 2018 Bossuet-Greif et al. 2018 Bossuet-Greif et al. This content is distributed under the terms of the Creative Commons Attribution 4.0 International license.

10.1128/mBio.02393-17.8 FIG S8  53BP1 and RPA are recruited in response to *pks*^+^ *E. coli*-induced DNA damage. (a) Representative images of RPA (green) and 53BP1 (red) immunostaining in HeLa cells infected for 4 h with DH10B pBAC*pks* or vector (MOI = 20) or treated for 4 h with 2.5 μM mitomycin C (MMC) and then washed and incubated with gentamicin for 4 or 20 h. DNA was counterstained with DAPI (blue). Scale bar = 20 μm. (b) Cells positive for RPA or 53BP1 were counted; means and standard deviations of results from at least three independent experiments are shown. Download FIG S8, TIF file, 1.7 MB. Copyright © 2018 Bossuet-Greif et al. 2018 Bossuet-Greif et al. This content is distributed under the terms of the Creative Commons Attribution 4.0 International license.

10.1128/mBio.02393-17.9 FIG S9  Inhibition of ATR abrogates FANCD2 monoubiquitinylation and focus formation in HeLa cells infected with *pks*^+^ *E. coli*. (a) HeLa cells were exposed for 4 h to *E. coli* DH10B hosting the BACpks or vector (MOI = 200) or treated with cisplatin (CPT). Then, ATRi was added or not and, following 8 h of incubation, the modification of FANCD2 was examined by Western blotting. The upper FANCD2 band was formed by monoubiquitination. (b) Representative images of p-H2AX and FANCD2 immunostaining in HeLa cells infected for 4 h (MOI = 20) or treated with CPT and then washed, treated or not with ATRi, and incubated for 20 h. DNA was counterstained with DAPI. Scale bar = 20 μm. Download FIG S9, TIF file, 2 MB. Copyright © 2018 Bossuet-Greif et al. 2018 Bossuet-Greif et al. This content is distributed under the terms of the Creative Commons Attribution 4.0 International license.

The recruitment of FANCD2 upon infection with *pks*^+^ *E. coli* argues for a role of the Fanconi anemia pathway in the management of ICLs induced by colibactin. Thus, we next asked whether inhibition of ICL repair results in hypersensitivity of the cells to colibactin. To test this hypothesis, FANCD2 expression was knocked down in HeLa cells using small interfering RNA (siRNA) (Fig. S10a), the cells were infected with DH10 pBAC*pks* or vector, and cell survival was assessed in a clonogenic assay. FANCD2-depleted cells formed significantly fewer colonies than HeLa cells transfected with a scrambled siRNA 7 days after infection with DH10B pBAC*pks* but not after infection with DH10B pBAC vector (Fig. 5d; see also Fig. S10b). Thus, loss of ICL repair sensitizes HeLa cells to colibactin-producing *E. coli*. Together, these data demonstrate that in human cells, the Fanconi pathway machinery that is central to ICL repair is recruited following colibactin-induced DNA damage and is required for survival of infection with colibactin-producing bacteria.

10.1128/mBio.02393-17.10 FIG S10  Knockdown of FANCD2 and HeLa cell clonogeny following infection with *pks*^+^ *E. coli*. (a) HeLa cells were transfected with scramble (control) or FANCD2 siRNA, and soluble cell extracts were prepared to assess FANCD2 protein levels by Western blotting. Lamin A is shown as a loading control. (b) HeLa cells were transfected with scrambled or FANCD2 siRNAs and then infected with *E. coli* DH10B hosting the BAC *pks* or vector (MOI = 12). Following a 4-h infection, the cells were washed and subjected to trypsinization, and 300 cells/well were seeded in 6-well plates and grown for 7 days. This assay measures the ability of individual cells to divide and form new colonies, allowing precise assessment of cell survival. The colonies formed by surviving cells were stained and photographed. Colonies containing at least 50 cells were counted. Clonogenic cell survival was corrected for plating efficiency by normalizing with the number of colonies formed by control cells. Download FIG S10, TIF file, 1.1 MB. Copyright © 2018 Bossuet-Greif et al. 2018 Bossuet-Greif et al. This content is distributed under the terms of the Creative Commons Attribution 4.0 International license.

### Colibactin generates DNA interstrand cross-links *in cellulo*.

**FIG 6:**
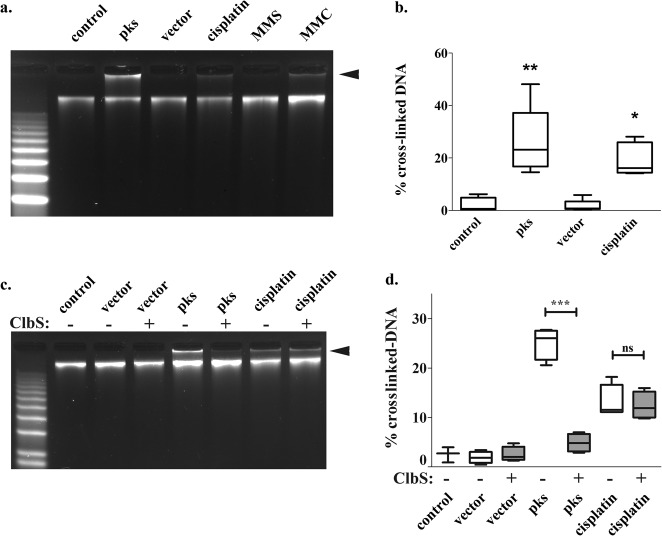
*In cellulo* DNA interstrand cross-links in the genomic DNA of HeLa cells infected with *pks*^+^ bacteria. (a) HeLa cells were exposed for 4 h to *E. coli* DH10B hosting the BAC *pks* gene or vector (MOI = 200) or were treated with 13 μM MMC, 80 μM cisplatin, or 250 µM MMS. Bacteria were removed by washing, and then HeLa cell genomic DNA was extracted and analyzed by denaturing electrophoresis. (b) The box-and-whisker plots represent the percentages (median, interquartile, and min/max values) of the DNA signal in the upper, cross-linked band (arrowhead in panel a) relative to the total DNA signal in the lane, as determined by image analysis in five independent experiments. *, *P* < 0.05; **, *P* < 0.01 (compared to control; one-way ANOVA with Bonferroni posttest). (c) HeLa cells were exposed for 4 h to *E. coli* DH10B hosting the BAC *pks* gene or vector (MOI = 200) or were treated with 80 μM cisplatin in the presence or absence of 400 nM purified ClbS protein. The bacteria were removed by washing, and cell genomic DNA was extracted and analyzed by denaturing electrophoresis. (d) Quantification in four independent experiments. ns, not significant; ***, *P* < 0.001 (one-way ANOVA with Bonferroni posttest).

To examine directly the occurrence of DNA ICLs in human cells exposed to *pks*^+^ enterobacteria, we inspected the cell genomic DNA by denaturing gel electrophoresis. HeLa cells were infected for 4 h with DH10B hosting pBAC*pks* or with empty vector. As controls, cells were treated for 4 h with MMS, MMC, or cisplatin. HeLa cell genomic DNA was extracted immediately at the end of the infection period and analyzed on denaturing agarose gels (Fig. 6a). As expected, the genomic DNA of untreated cells migrated in the electrophoretic gel as an intact band with a high apparent molecular weight. In contrast, because ICLs prevent the denaturation of DNA and therefore inhibit its electrophoretic migration (47), a significant fraction of the genomic DNA of the cells treated with an ICL-inducing agent (cisplatin or MMC), but not of those treated with the monofunctional alkylating agent MMS, did not enter the gel and remained in the loading well (Fig. 6a and b). Similarly, the genomic DNA of cells infected with DH10B pBAC*pks* exhibited a significant fraction of cross-linked DNA that did not migrate (Fig. 6a and b). This result indicated that DNA cross-links are rapidly generated by colibactin in infected HeLa cells. The addition of purified ClbS protein during the infection reduced the fraction of nonmigrating DNA in cells infected with *pks*^+^ bacteria to background levels but not in cisplatin-treated cells, thus confirming the involvement of the natural mature colibactin (Fig. 6c and d). Together with the replication stress and recruitment of the Fanconi anemia ICL repair pathway in *pks*-infected cells, these results demonstrate that the natural colibactin genotoxin generates DNA ICL in infected human cells.

## DISCUSSION

In this study, we addressed the issue of the mode of action on DNA and resulting genotoxicity in the host cell of the natural colibactins produced by *pks*^+^ enterobacteria. As natural active colibactins resist isolation, we used live bacteria with an intact colibactin synthesis pathway, under culture conditions that allowed production of the genotoxic metabolites as demonstrated by the genotoxicity for the infected epithelial cells. In summary, we observed that addition of exogenous bacterial or mammalian DNA during the infection of epithelial cells by *pks*^+^ *E. coli* protected the cells from colibactin genotoxicity. The DNA directly exposed to colibactin-producing bacteria did not exhibit strand breaks but did exhibit cross-linking of the DNA strands. The *in vitro* cross-linking activity of *pks*^+^ *E. coli* was also observed on naked DNA in the absence of epithelial cells. This DNA cross-linking was similar to that induced by MMC and cisplatin (a natural cytotoxin and a synthetic antineoplastic drug, respectively), which are bifunctional DNA alkylators generating ICLs. All *pks*^+^ enterobacterial species (including clinical *E. coli*, *C. koseri*, and *K. pneumoniae* isolates) exerted the same phenotype. Mutagenesis of genes inactivating (pre)colibactin synthesis or inactivating the deacylation step that maturates precolibactins into genotoxic colibactins resulted in a complete loss of the cross-linking activity. These *in vitro* results strongly argue for a direct interaction of the natural colibactins with double-stranded DNA and for covalent cross-linking of the two complementary strands, resulting in DNA ICLs. Such a mode of action is in good agreement with the structural features that were recently predicted or identified in candidate nongenotoxic precolibactins: a thiazoline-thiazole tail and a spirocyclic cyclopropane ring (48, 49). Thiazole rings have been shown to allow DNA intercalation of DNA-targeting natural products such as the bleomycins, thus supporting the idea of a direct interaction of colibactins with DNA. Cyclopropane “warheads” are found in potent DNA-damaging natural products such as illudins, CC-1065, yatakemycin, and duocarmycins, which alkylate DNA through nucleophilic cyclopropane ring openings. An isolated candidate precolibactin (purified from a nongenotoxic *clbP* mutant) bearing the cyclopropane showed weak cross-linking activity when added in millimolar amounts to plasmid DNA and incubated for 20 h (32). In addition, synthetic compounds bearing a cyclopropane ring system similar to that detected in precolibactin candidates from *clbP* mutants were recently shown to alkylate plasmid DNA, resulting in extensive DNA cleavage (but not cross-linking) (33). The NRPS ClbH incorporates the cyclopropane during synthesis of (pre)colibactins, and we observed that *clbH* gene inactivation fully abrogated the DNA damage in infected cells and the cross-linking of plasmid DNA *in vitro*. In addition, both the DNA cross-linking and genotoxicity of *pks*^+^ *E. coli* were inhibited by addition of purified self-resistance protein ClbS, which was recently shown to convert the cyclopropane into inactive products by hydrolysis (27). Altogether, these data support a model where the natural colibactins directly damage double-stranded DNA, with a key role for the electrophilic cyclopropane in the alkylation of one DNA strand, formation of a second electrophilic site, and alkylation of the cDNA strand, resulting in an ICL.

Denaturing gel electrophoresis of DNA from epithelial cells infected with *pks*^+^ *E. coli* indicated that ICLs are rapidly (as soon as the end of the 4-h infection) generated within host cell genomic DNA. Thus, colibactins produced by *pks*^+^ *E. coli* not only directly damage and cross-link naked DNA *in vitro* but also can traffic in the infected epithelial cell, reach the nucleus, and cross-link DNA that is packaged into chromatin. DNA ICLs are recognized by the cellular DNA damage response mainly during DNA replication, halting the replication machinery and generating patches of single-stranded DNA (50, 51). ATR is activated in response to this replication stress signaled by the patches of single-stranded DNA directly bound by RPA (39). A previously published observation that *pks*^+^ *E. coli*-exposed cells lagged 48 h in the S phase before accumulating in the G_2_ phase whereas control cells proceeded through S-G_2_-M phases in 12 h (1) is consistent with the occurrence of replication stress. The recruitment of RPA in subnuclear foci and the activation/phosphorylation of ATR and its downstream substrate CHK1 also indicate the induction of replication stress in cells exposed to *pks*^+^ *E. coli*. Moreover, ATR activation was central in orchestrating the cellular response to colibactin-induced damage, since its pharmacological inhibition abrogated the DNA damage response and resulted in mitotic catastrophe and cell death. Activated ATR allowed the recruitment of the Fanconi anemia DNA cross-link repair pathway, evidenced by the activation of FANCD2 and its recruitment in subnuclear foci together with p-H2AX. As for ATR, FANCD2 was required for cell survival of colibactin-induced damage, as shown by the decreased level clonogeny upon FANCD2 depletion. Thus, induction of early genomic DNA ICLs by colibactins resulted in replication stress and activation of the ATR and Fanconi anemia repair pathways. Concurrently, DNA DSBs were formed, evidenced by ATM phosphorylation and the recruitment of p-H2AX and 53BP1 in subnuclear repair foci. Colibactin-induced ICLs are thus converted into DNA DSBs during the repair process, when DNA lesions are excised by the host repair nucleases (52, 53). Additionally, extended replication stalling and fork collapse can result in aberrant DNA structures and DSBs (54). The DNA-colibactin adducts might also degrade, as observed *in vitro* with synthetic colibactins mimics (33), or under oxidative conditions, colibactins could form reactive peroxides, leading to further DNA damage (27). We also cannot exclude the possibility that other types of DNA insults might be inflicted by different forms of colibactins, leading to DSBs by distinct mechanisms.

In conclusion, to our knowledge, this represents the first report of a direct induction of DNA ICLs in the mammalian genome by a bacterial infection. Such lesions induce replication stress and favor DSBs, and it has been shown that their accumulation over time contributes to aging in tissue and to genomic instability, a hallmark of precancerous cells. ICL is also an extremely lethal form of DNA damage, as it poses an unsurpassable block to replication and suppresses other fundamental DNA processes such as transcription and maintenance. Thus, these findings delineate a role of colibactins in tumorigenesis that might be more complex than previously thought.

## MATERIALS AND METHODS

### Bacterial strains, isogenic mutants, and cultures.

The *E. coli* strains used in this study were DH10B hosting the pBACpks or the vector (pBeloBAC11) (1). The *clbA* and *clbP* mutants, and the mutants complemented with the corresponding plasmids pClbA and pClbP, were described previously (23, 28). The *clbH* and *clbQ* mutants were constructed by using the lambda Red recombinase method, with primers AS149 and AS150, and primers IHAPJPN57 and IHAPJPN58. The corresponding complementation plasmids were constructed by cloning into pASK75 (SacI/PstI) the PCR products with primers CPTPNG14 and CPTPNG15 and primers CPTPNG20 and CPTPNG21. The primer sequences can be requested from the corresponding author. The wild-type clinical enterobacterial isolates were *E. coli* strain SP15 (28), *C. koseri* strain BAA 895, *K. pneumoniae* strain CF1, and *E. aerogenes* strain 64 (2). Bacteria were grown at 37°C in 5 ml of Lennox L broth (LB; Invitrogen) or on LB agar plates. Carbenicillin (50 µg ⋅ ml^−1^), kanamycin (50 µg ⋅ ml^−1^), or chloramphenicol (25 µg ⋅ ml^−1^) was added as required. For DNA cross-linking and HeLa cell infection experiments, bacteria were grown overnight in LB and then diluted 1/20 in prewarmed Dulbecco’s modified Eagle’s medium (DMEM)–25 mM HEPES (Invitrogen) and incubated at 37°C with 240-rpm agitation to reach the exponential phase (optical density at 600 nm [OD_600_], 0.4 to 0.5).

### *In vitro* DNA cross-linking assay.

Plasmid pUC19 DNA linearized with BamHI (TaKaRa) and purified with a QIAquick PCR kit (Qiagen) was stored at −20°C. Calf thymus DNA (Sigma) solubilized in 10 mM Tris–1 mM EDTA (pH 8) was sonicated to reduce the average DNA fragment size to ~1,000 bp and was stored at +4°C. Plasmid or thymus DNA was exposed to bacteria as follows: in a 96-well plate or in 1.5-ml Eppendorf microtubes, 400 ng (or the indicated amount) of DNA was added to 100 µl DMEM–25 mM HEPES (Invitrogen) inoculated with 3 × 10^6^ bacteria pregrown to the exponential phase or in the population numbers given in the text. As controls, DNA was treated with 5 mM MMS (Sigma), 80 µM cisplatin (Sigma), or 150 µM MMC (Sigma) activated with 5 mM dithiothreitol (DTT). Following 4 h at 37°C, bacteria were pelleted and the DNA was purified from the supernatant using a Qiagen QIAquick PCR kit. For wild-type enterobacterial strains that release nucleases, the bacteria were grown in DMEM–25 mM HEPES without the target DNA for 3.5 h, and then EDTA (1 mM) and target DNA were added and the mixture was incubated for 40 min at 37°C.

### Denaturing gel DNA electrophoresis.

The protocol was adapted from reference 55. Briefly, 1% agarose gels prepared in 100 mM NaCl–2 mM EDTA (pH 8) solution were soaked in 40 mM NaOH–1 mM EDTA electrophoresis running buffer. DNA was loaded with blue or violet gel loading dye (NEB), and then electrophoresis was carried out at room temperature for 45 min at 1 V/cm and then for 2 h at 2 V/cm. Following neutralization in 150 mM NaCl–100 mM Tris (pH 7.4), DNA was stained with Gel Red (Biotium) and visualized with flat-fielding while avoiding charge-coupled-device (CCD) pixel saturation in a Bio-Rad Chemidoc XRS system.

### Denaturation-renaturation assay.

The experiment was performed as previously described (35). Briefly, 100 ng of DNA was added to a mixture of 250 µl of 0.5 µg/ml ethidium bromide (Sigma), 0.4 mM EDTA, and 20mM potassium phosphate (pH 11.7). The fluorescence was measured with a Tecan Infinite Pro microplate reader (excitation wavelength, 525 nm; emission wavelength, 600 nm), and then the samples were heated for 3 min at 96°C and immediately cooled to 22°C, and the fluorescence was measured again. The percentage of DNA renaturation was calculated as the value corresponding to the level of fluorescence measured after denaturation and renaturation divided by the value corresponding to the level of fluorescence measured before denaturation.

### HeLa cell culture, infection, and *in cellulo* cross-linking assay.

HeLa cells (ATCC CCL2) were cultivated in DMEM Glutamax supplemented with 10% fetal calf serum (FCS) and 1% nonessential amino acids (Invitrogen) in a 37°C 5% CO_2_ incubator and were maintained by serial passage. Cell infections were performed as described previously (1). Briefly, 1.5 × 10^4^ cells/well were seeded in 96-well cell culture plates and grown during 24 h. Cells were washed, placed in DMEM–25 mM HEPES medium (Invitrogen), and inoculated with bacteria at the given multiplicity of infection (MOI [the number of bacteria per cell at the onset of infection]). Extracellular double-stranded DNA was added at the indicated concentrations. After a 4-h infection at 37°C, the cells were washed and incubated for 4 h in complete cell culture medium supplemented with 100 µg/ml gentamicin. To examine DNA interstrand cross-links in HeLa cells, 3 × 10^5^ cells/well were seeded in 6-well plates and grown 24 h. Cells were infected during 4 h (MOI = 200) and then washed and immediately collected by trypsinization. Control cells were treated for 4 h with 80 µM cisplatin, with 13 µM MMC supplemented with 5 mM DTT, or with 250 μM MMS. HeLa cell genomic DNA was purified with a Qiagen DNeasy blood and tissue kit.

### Phospho-H2AX quantification and immunofluorescence analyses.

HeLa cells were grown and placed on 96-well plates or 12-well chamber slides. The quantification of p-H2AX was performed as described before (26). Briefly, following fixation with 4% formaldehyde, permeabilization, and blocking, cells were incubated overnight at 4°C with anti-S139-phosphorylated H2AX antibodies (Cell Signaling catalog no. 9718). An infrared fluorescent (800-nm-wavelength) secondary antibody (Rockland) was used to detect p-H2AX. DNA was counterstained with RedDot2 (Biotium). DNA and p-H2AX were visualized with an Odyssey infrared imaging scanner (Li-Cor Biosciences). The values representing relative levels of fluorescent units for p-H2AX per cell (as determined by dividing the p-H2AX signal by the DNA signal) were divided by the control values to determine the genotoxic index values.

Immunofluorescence analyses for RPA, 53BP1, FANCD2, and S139-phosphorylated H2AX were performed as previously described (56). Briefly, following preextraction with phosphate-buffered saline (PBS)-0.1% Triton X-100, fixation with 4% formaldehyde, and permeabilization in 0.5% Triton X-100, the cells were stained with the following primary antibodies: 53BP1 (NB100-304) and FANCD2 (NB100-182) antibodies from Novus Biologicals, Inc., p-H2AX (JBW301) from Merck/Millipore, and RPA32 (NA18) from Calbiochem. Cells were washed and incubated with the secondary antibodies rhodamine Red X (R6394) and Alexa Fluor 488 anti-mouse antibody (A11017) or anti-rabbit antibody (A11070) (Invitrogen). DNA was counterstained with DAPI (4′,6-diamidino-2-phenylindole). Cells were examined with an epifluorescence microscope (Nikon 50i) and counted as positive for focus formation when >10 foci/nucleus were detected.

### Western blotting.

Cells were washed and lysed directly in the cell culture well in 2× Laemmli sample buffer (Bio-Rad). The cell lysates were then sonicated and heated for 10 min at 70°C, and aliquots were stored at −20°C. Proteins were separated by SDS-PAGE and transferred to nitrocellulose membranes (Amersham). Membranes were incubated with the primary ATM (2873), p-ATM (5883), p-ATR, Chk1 (2360), and p-Chk1 (2348) antibodies from Cell Signaling; p-RPA32 (A300-246) antibody from Bethyl; and lamin A/C (SAB4200236) and ATR (SAB4200348) antibodies from Sigma. Secondary anti-mouse or anti-rabbit horseradish peroxidase (HRP)-conjugated antibodies (Jackson Laboratories) were visualized with chemiluminescence Clarity Western ECL substrate (Bio-Rad) and imaged using a ChemiDoc XRS imager and Image Lab software (Bio-Rad).

### ATR inhibition, RNA interference, and survival assay.

ATR inhibitor VE-821 (Selleckchem) was used at a final concentration of 10 μM. After 48 h of incubation, cell survival was quantified by the use of a colorimetric assay (57). Briefly, cells were fixed and stained with methylene blue, and then the stain was extracted with 0.1 M HCl and quantified by an optical density reading at 660 nm using a Tecan microplate reader.

Gene silencing of HeLa cells was performed by transfection of siRNA (Sigma) using INTERFERin (Polyplus) as described before (56). Briefly, HeLa cells were transfected with scrambled (CAUGUCAUGUGUCACAUCU-dTdT) (where "dTdT" represents deoxythymidine dinucleotide) or FANCD2 (AACAGCCAUGGAUACACUUGA-dTdT) siRNA. After 48 h of incubation, cells were replated in 96- or 6-well plates for further treatments at 72 h posttransfection. Clonogenic cell survival was assessed using colony formation (58).

### ClbS protein purification.

BL21(DE3) cells hosting the plasmid pET28a-ClbS-His (27) were grown overnight at 37°C in 50 ml LB–0.4 mM IPTG (isopropyl-β-d-thiogalactopyranoside). Bacteria were lysed by sonication in a mixture containing 50 mM NaH_2_PO_4_, 300 mM NaCl, 40 mM imidazole (pH 8), 200 μg/ml gentamicin, 1 mg/ml lysozyme, and complete protease inhibitor cocktail (Sigma). The cleared lysate was incubated for 1 h with HisPur nickel-nitrilotriacetic acid (Ni-NTA) agarose (Thermo Scientific) in lysis buffer, washed with a mixture containing 50 mM NaH_2_PO_4_, 300 mM NaCl, and 60 mM imidazole (pH 8), and eluted in 250 mM imidazole buffer. The eluate was stored at +4°C before use at 400 nM in a 100-μl or 2.5-ml volume for culture experiments.

### Statistical analyses.

Statistical analyses were carried out using GraphPad Prism 5.0b. *P* values were calculated using analysis of variance (ANOVA) followed by Bonferroni or Tukey posttests.

## References

[1] Nougayrède JP, Homburg S, Taieb F, Boury M, Brzuszkiewicz E, Gottschalk G, Buchrieser C, Hacker J, Dobrindt U, Oswald E 2006 Escherichia coli induces DNA double-strand breaks in eukaryotic cells. Science 313:848–851. doi:10.1126/science.1127059. 16902142

[2] Putze J, Hennequin C, Nougayrède JP, Zhang W, Homburg S, Karch H, Bringer MA, Fayolle C, Carniel E, Rabsch W, Oelschlaeger TA, Oswald E, Forestier C, Hacker J, Dobrindt U 2009 Genetic structure and distribution of the colibactin genomic island among members of the family Enterobacteriaceae. Infect Immun 77:4696–4703. doi:10.1128/IAI.00522-09. 19720753 PMC2772509

[3] Bondarev V, Richter M, Romano S, Piel J, Schwedt A, Schulz-Vogt HN 2013 The genus Pseudovibrio contains metabolically versatile bacteria adapted for symbiosis. Environ Microbiol 15:2095–2113. doi:10.1111/1462-2920.12123. 23601235 PMC3806328

[4] Engel P, Vizcaino MI, Crawford JM 2015 Gut symbionts from distinct hosts exhibit genotoxic activity via divergent colibactin biosynthesis pathways. Appl Environ Microbiol 81:1502–1512. doi:10.1128/AEM.03283-14. 25527542 PMC4309719

[5] Moretti C, Hosni T, Vandemeulebroecke K, Brady C, De Vos P, Buonaurio R, Cleenwerck I 2011 Erwinia oleae sp. nov., isolated from olive knots caused by Pseudomonas savastanoi pv. savastanoi. Int J Syst Evol Microbiol 61:2745–2752. doi:10.1099/ijs.0.026336-0. 21186287

[6] Johnson JR, Johnston B, Kuskowski MA, Nougayrede JP, Oswald E 2008 Molecular epidemiology and phylogenetic distribution of the Escherichia coli pks genomic island. J Clin Microbiol 46:3906–3911. doi:10.1128/JCM.00949-08. 18945841 PMC2593299

[7] Krieger JN, Dobrindt U, Riley DE, Oswald E 2011 Acute Escherichia coli prostatitis in previously health young men: bacterial virulence factors, antimicrobial resistance, and clinical outcomes. Urology 77:1420–1425. doi:10.1016/j.urology.2010.12.059. 21459419

[8] Chen YT, Lai YC, Tan MC, Hsieh LY, Wang JT, Shiau YR, Wang HY, Lin AC, Lai JF, Huang IW, Lauderdale TL 2017 Prevalence and characteristics of pks genotoxin gene cluster-positive clinical Klebsiella pneumoniae isolates in Taiwan. Sci Rep 7:43120. doi:10.1038/srep43120. 28233784 PMC5324043

[9] Marcq I, Martin P, Payros D, Cuevas-Ramos G, Boury M, Watrin C, Nougayrède JP, Olier M, Oswald E 2014 The genotoxin colibactin exacerbates lymphopenia and decreases survival rate in mice infected with septicemic Escherichia coli. J Infect Dis 210:285–294. doi:10.1093/infdis/jiu071. 24489107

[10] McCarthy AJ, Martin P, Cloup E, Stabler RA, Oswald E, Taylor PW 2015 The genotoxin colibactin is a determinant of virulence in Escherichia coli K1 experimental neonatal systemic infection. Infect Immun 83:3704–3711. doi:10.1128/IAI.00716-15. 26150540 PMC4534652

[11] Lu MC, Chen YT, Chiang MK, Wang YC, Hsiao PY, Huang YJ, Lin CT, Cheng CC, Liang CL, Lai YC 2017 Colibactin contributes to the hypervirulence of pks(+) K1 CC23 Klebsiella pneumoniae in mouse meningitis infections. Front Cell Infect Microbiol 7:103. doi:10.3389/fcimb.2017.00103. 28409125 PMC5374149

[12] Nowrouzian FL, Oswald E 2012 Escherichia coli strains with the capacity for long-term persistence in the bowel microbiota carry the potentially genotoxic pks island. Microb Pathog 53:180–182. doi:10.1016/j.micpath.2012.05.011. 22709536

[13] Arthur JC, Perez-Chanona E, Mühlbauer M, Tomkovich S, Uronis JM, Fan TJ, Campbell BJ, Abujamel T, Dogan B, Rogers AB, Rhodes JM, Stintzi A, Simpson KW, Hansen JJ, Keku TO, Fodor AA, Jobin C 2012 Intestinal inflammation targets cancer-inducing activity of the microbiota. Science 338:120–123. doi:10.1126/science.1224820. 22903521 PMC3645302

[14] Buc E, Dubois D, Sauvanet P, Raisch J, Delmas J, Darfeuille-Michaud A, Pezet D, Bonnet R 2013 High prevalence of mucosa-associated E. coli producing cyclomodulin and genotoxin in colon cancer. PLoS One 8:e56964. doi:10.1371/journal.pone.0056964. 23457644 PMC3572998

[15] Bonnet M, Buc E, Sauvanet P, Darcha C, Dubois D, Pereira B, Déchelotte P, Bonnet R, Pezet D, Darfeuille-Michaud A 2014 Colonization of the human gut by E. coli and colorectal cancer risk. Clin Cancer Res 20:859–867. doi:10.1158/1078-0432.CCR-13-1343. 24334760

[16] Cougnoux A, Dalmasso G, Martinez R, Buc E, Delmas J, Gibold L, Sauvanet P, Darcha C, Déchelotte P, Bonnet M, Pezet D, Wodrich H, Darfeuille-Michaud A, Bonnet R 2014 Bacterial genotoxin colibactin promotes colon tumour growth by inducing a senescence-associated secretory phenotype. Gut 63:1932–1942. doi:10.1136/gutjnl-2013-305257. 24658599

[17] Tomkovich S, Yang Y, Winglee K, Gauthier J, Mühlbauer M, Sun X, Mohamadzadeh M, Liu X, Martin P, Wang GP, Oswald E, Fodor AA, Jobin C 2017 Locoregional effects of microbiota in a preclinical model of colon carcinogenesis. Cancer Res 77:2620–2632. doi:10.1158/0008-5472.CAN-16-3472. 28416491 PMC5468752

[18] Martin P, Marcq I, Magistro G, Penary M, Garcie C, Payros D, Boury M, Olier M, Nougayrède JP, Audebert M, Chalut C, Schubert S, Oswald E 2013 Interplay between siderophores and colibactin genotoxin biosynthetic pathways in Escherichia coli. PLoS Pathog 9:e1003437. doi:10.1371/journal.ppat.1003437. 23853582 PMC3708854

[19] Beld J, Sonnenschein EC, Vickery CR, Noel JP, Burkart MD 2014 The phosphopantetheinyl transferases: catalysis of a post-translational modification crucial for life. Nat Prod Rep 31:61–108. doi:10.1039/c3np70054b. 24292120 PMC3918677

[20] Li ZR, Li J, Gu JP, Lai JYH, Duggan BM, Zhang WP, Li ZL, Li YX, Tong RB, Xu Y, Lin DH, Moore BS, Qian PY 2016 Divergent biosynthesis yields a cytotoxic aminomalonate-containing precolibactin. Nat Chem Biol 12:773–775. doi:10.1038/nchembio.2157. 27547923 PMC5030165

[21] Guntaka NS, Healy AR, Crawford JM, Herzon SB, Bruner SD 2017 Structure and functional analysis of ClbQ, an unusual intermediate-releasing thioesterase from the colibactin biosynthetic pathway. ACS Chem Biol 12:2598–2608. doi:10.1021/acschembio.7b00479. 28846367 PMC5830302

[22] Mousa JJ, Yang Y, Tomkovich S, Shima A, Newsome RC, Tripathi P, Oswald E, Bruner SD, Jobin C 2016 MATE transport of the E. coli-derived genotoxin colibactin. Nat Microbiol 1:15009. doi:10.1038/nmicrobiol.2015.9. 27571755 PMC5704960

[23] Dubois D, Baron O, Cougnoux A, Delmas J, Pradel N, Boury M, Bouchon B, Bringer MA, Nougayrède JP, Oswald E, Bonnet R 2011 ClbP is a prototype of a peptidase subgroup involved in biosynthesis of nonribosomal peptides. J Biol Chem 286:35562–35570. doi:10.1074/jbc.M111.221960. 21795676 PMC3195562

[24] Brotherton CA, Balskus EP 2013 A prodrug resistance mechanism is involved in colibactin biosynthesis and cytotoxicity. J Am Chem Soc 135:3359–3362. doi:10.1021/ja312154m. 23406518

[25] Bian X, Fu J, Plaza A, Herrmann J, Pistorius D, Stewart AF, Zhang Y, Müller R 2013 In vivo evidence for a prodrug activation mechanism during colibactin maturation. Chembiochem 14:1194–1197. doi:10.1002/cbic.201300208. 23744512

[26] Bossuet-Greif N, Dubois D, Petit C, Tronnet S, Martin P, Bonnet R, Oswald E, Nougayrède JP 2016 Escherichia coli ClbS is a colibactin resistance protein. Mol Microbiol 99:897–908. doi:10.1111/mmi.13272. 26560421

[27] Tripathi P, Shine EE, Healy AR, Kim CS, Herzon SB, Bruner SD, Crawford JM 2017 ClbS is a cyclopropane hydrolase that confers colibactin resistance. J Am Chem Soc 139:17719–17722. 29112397 10.1021/jacs.7b09971 PMC6202678

[28] Cuevas-Ramos G, Petit CR, Marcq I, Boury M, Oswald E, Nougayrède JP 2010 Escherichia coli induces DNA damage in vivo and triggers genomic instability in mammalian cells. Proc Natl Acad Sci U S A 107:11537–11542. doi:10.1073/pnas.1001261107. 20534522 PMC2895108

[29] Secher T, Samba-Louaka A, Oswald E, Nougayrède JP 2013 Escherichia coli producing colibactin triggers premature and transmissible senescence in mammalian cells. PLoS One 8:e77157. doi:10.1371/journal.pone.0077157. 24116215 PMC3792898

[30] Payros D, Secher T, Boury M, Brehin C, Ménard S, Salvador-Cartier C, Cuevas-Ramos G, Watrin C, Marcq I, Nougayrède JP, Dubois D, Bedu A, Garnier F, Clermont O, Denamur E, Plaisancié P, Theodorou V, Fioramonti J, Olier M, Oswald E 2014 Maternally acquired genotoxic Escherichia coli alters offspring’s intestinal homeostasis. Gut Microbes 5:313–325. doi:10.4161/gmic.28932. 24971581 PMC4153768

[31] Lai YC, Lin AC, Chiang MK, Dai YH, Hsu CC, Lu MC, Liau CY, Chen YT 2014 Genotoxic Klebsiella pneumoniae in Taiwan. PLoS One 9:e96292. doi:10.1371/journal.pone.0096292. 24852749 PMC4031060

[32] Vizcaino MI, Crawford JM 2015 The colibactin warhead crosslinks DNA. Nat Chem 7:411–417. doi:10.1038/nchem.2221. 25901819 PMC4499846

[33] Healy AR, Nikolayevskiy H, Patel JR, Crawford JM, Herzon SB 2016 A mechanistic model for Colibactin-induced genotoxicity. J Am Chem Soc 138:15563–15570. doi:10.1021/jacs.6b10354. 27934011 PMC5359767

[34] Rogakou EP, Pilch DR, Orr AH, Ivanova VS, Bonner WM 1998 DNA double-stranded breaks induce histone H2AX phosphorylation on serine 139. J Biol Chem 273:5858–5868. doi:10.1074/jbc.273.10.5858. 9488723

[35] Lown JW, Begleiter A, Johnson D, Morgan AR 1976 Studies related to antitumor antibiotics. Part V. Reactions of mitomycin C with DNA examined by ethidium fluorescence assay. Can J Biochem 54:110–119. doi:10.1139/o76-018. 4201

[36] Reuter C, Alzheimer M, Walles H, Oelschlaeger TA 2018 An adherent mucus layer attenuates the genotoxic effect of colibactin. Cell Microbiol 20. doi:10.1111/cmi.12812. 29156489

[37] Zha L, Jiang Y, Henke MT, Wilson MR, Wang JX, Kelleher NL, Balskus EP 2017 Colibactin assembly line enzymes use S-adenosylmethionine to build a cyclopropane ring. Nat Chem Biol 13:1063–1065. doi:10.1038/nchembio.2448. 28805802 PMC5657534

[38] Räschle M, Knipscheer P, Enoiu M, Angelov T, Sun J, Griffith JD, Ellenberger TE, Schärer OD, Walter JC 2008 Mechanism of replication-coupled DNA interstrand crosslink repair. Cell 134:969–980. doi:10.1016/j.cell.2008.08.030. 18805090 PMC2748255

[39] Saldivar JC, Cortez D, Cimprich KA 2017 The essential kinase ATR: ensuring faithful duplication of a challenging genome. Nat Rev Mol Cell Biol 18:622–636. doi:10.1038/nrm.2017.67. 28811666 PMC5796526

[40] Vassin VM, Anantha RW, Sokolova E, Kanner S, Borowiec JA 2009 Human RPA phosphorylation by ATR stimulates DNA synthesis and prevents ssDNA accumulation during DNA-replication stress. J Cell Sci 122:4070–4080. doi:10.1242/jcs.053702. 19843584 PMC2776501

[41] Reaper PM, Griffiths MR, Long JM, Charrier JD, Maccormick S, Charlton PA, Golec JMC, Pollard JR 2011 Selective killing of ATM- or p53-deficient cancer cells through inhibition of ATR. Nat Chem Biol 7:428–430. doi:10.1038/nchembio.573. 21490603

[42] Castedo M, Perfettini JL, Roumier T, Andreau K, Medema R, Kroemer G 2004 Cell death by mitotic catastrophe: a molecular definition. Oncogene 23:2825–2837. doi:10.1038/sj.onc.1207528. 15077146

[43] Michl J, Zimmer J, Tarsounas M 2016 Interplay between Fanconi anemia and homologous recombination pathways in genome integrity. EMBO J 35:909–923. doi:10.15252/embj.201693860. 27037238 PMC4865030

[44] Montes de Oca R, Andreassen PR, Margossian SP, Gregory RC, Taniguchi T, Wang X, Houghtaling S, Grompe M, D’Andrea AD 2005 Regulated interaction of the Fanconi anemia protein, FANCD2, with chromatin. Blood 105:1003–1009. doi:10.1182/blood-2003-11-3997. 15454491

[45] Taniguchi T, Garcia-Higuera I, Andreassen PR, Gregory RC, Grompe M, D’Andrea AD 2002 S-phase-specific interaction of the Fanconi anemia protein, FANCD2, with BRCA1 and RAD51. Blood 100:2414–2420. doi:10.1182/blood-2002-01-0278. 12239151

[46] Andreassen PR, D’Andrea AD, Taniguchi T 2004 ATR couples FANCD2 monoubiquitination to the DNA-damage response. Genes Dev 18:1958–1963. doi:10.1101/gad.1196104. 15314022 PMC514175

[47] Rothfuss A, Grompe M 2004 Repair kinetics of genomic interstrand DNA cross-links: evidence for DNA double-strand break-dependent activation of the Fanconi anemia/BRCA pathway. Mol Cell Biol 24:123–134. doi:10.1128/MCB.24.1.123-134.2004. 14673148 PMC303365

[48] Balskus EP 2015 Colibactin: understanding an elusive gut bacterial genotoxin. Nat Prod Rep 32:1534–1540. doi:10.1039/c5np00091b. 26390983

[49] Healy AR, Herzon SB 2017 Molecular basis of gut microbiome-associated colorectal cancer: a synthetic perspective. J Am Chem Soc 139:14817–14824. doi:10.1021/jacs.7b07807. 28949546 PMC7024635

[50] Byun TS, Pacek M, Yee MC, Walter JC, Cimprich KA 2005 Functional uncoupling of MCM helicase and DNA polymerase activities activates the ATR-dependent checkpoint. Genes Dev 19:1040–1052. doi:10.1101/gad.1301205. 15833913 PMC1091739

[51] Deans AJ, West SC 2011 DNA interstrand crosslink repair and cancer. Nat Rev Cancer 11:467–480. doi:10.1038/nrc3088. 21701511 PMC3560328

[52] Hanada K, Budzowska M, Modesti M, Maas A, Wyman C, Essers J, Kanaar R 2006 The structure-specific endonuclease Mus81-Eme1 promotes conversion of interstrand DNA crosslinks into double-strands breaks. EMBO J 25:4921–4932. doi:10.1038/sj.emboj.7601344. 17036055 PMC1618088

[53] Clauson C, Schärer OD, Niedernhofer L 2013 Advances in understanding the complex mechanisms of DNA interstrand cross-link repair. Cold Spring Harb Perspect Biol 5:a012732. doi:10.1101/cshperspect.a012732. 24086043 PMC4123742

[54] Cimprich KA, Cortez D 2008 ATR: an essential regulator of genome integrity. Nat Rev Mol Cell Biol 9:616–627. doi:10.1038/nrm2450. 18594563 PMC2663384

[55] Cech TR 1981 Alkaline gel electrophoresis of deoxyribonucleic acid photoreacted with trimethylpsoralen: rapid and sensitive detection of interstrand cross-links. Biochemistry 20:1431–1437. doi:10.1021/bi00509a005. 6261794

[56] Bezine E, Malaisé Y, Loeuillet A, Chevalier M, Boutet-Robinet E, Salles B, Mirey G, Vignard J 2016 Cell resistance to the cytolethal distending toxin involves an association of DNA repair mechanisms. Sci Rep 6:36022. doi:10.1038/srep36022. 27775089 PMC5075911

[57] De Rycke J, Mazars P, Nougayrede JP, Tasca C, Boury M, Herault F, Valette A, Oswald E 1996 Mitotic block and delayed lethality in HeLa epithelial cells exposed to Escherichia coli BM2-1 producing cytotoxic necrotizing factor type 1. Infect Immun 64:1694–1705. 8613380 10.1128/iai.64.5.1694-1705.1996 PMC173981

[58] Franken NAP, Rodermond HM, Stap J, Haveman J, van Bree C 2006 Clonogenic assay of cells in vitro. Nat Protoc 1:2315–2319. doi:10.1038/nprot.2006.339. 17406473

