# Supplementary figures and images for: The Colibactin Genotoxin Generates DNA Interstrand Cross-Links in Infected Cells
Source: mBio. 2018 Mar 20;9(2):e02393-17. doi: 10.1128/mBio.02393-17 (PMC5874909; doi:10.1128/mBio.02393-17)

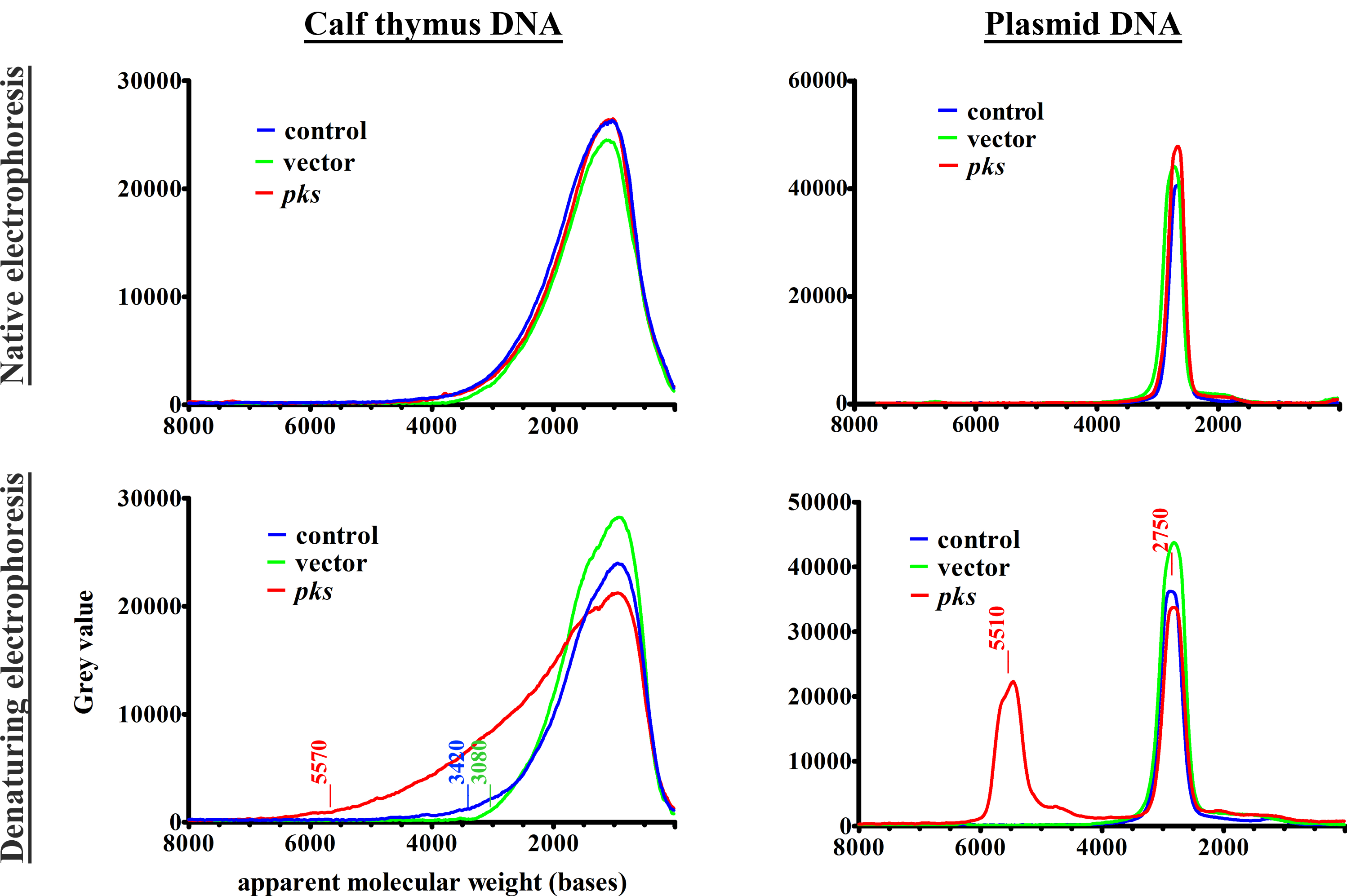

Supplement: FIG S1 [file mbo002183800sf1.tif]

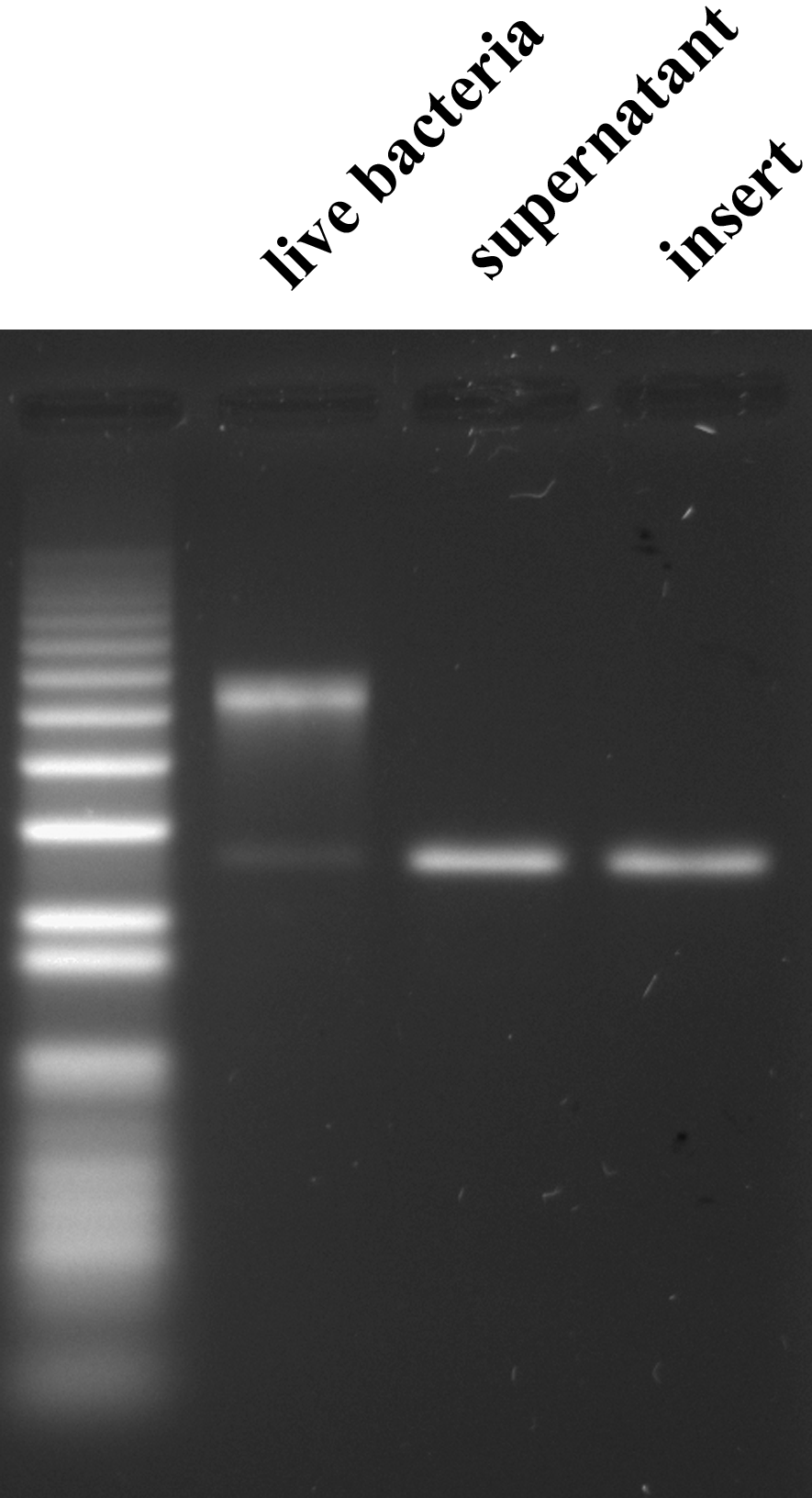

Supplement: FIG S2 [file mbo002183800sf2.tif]

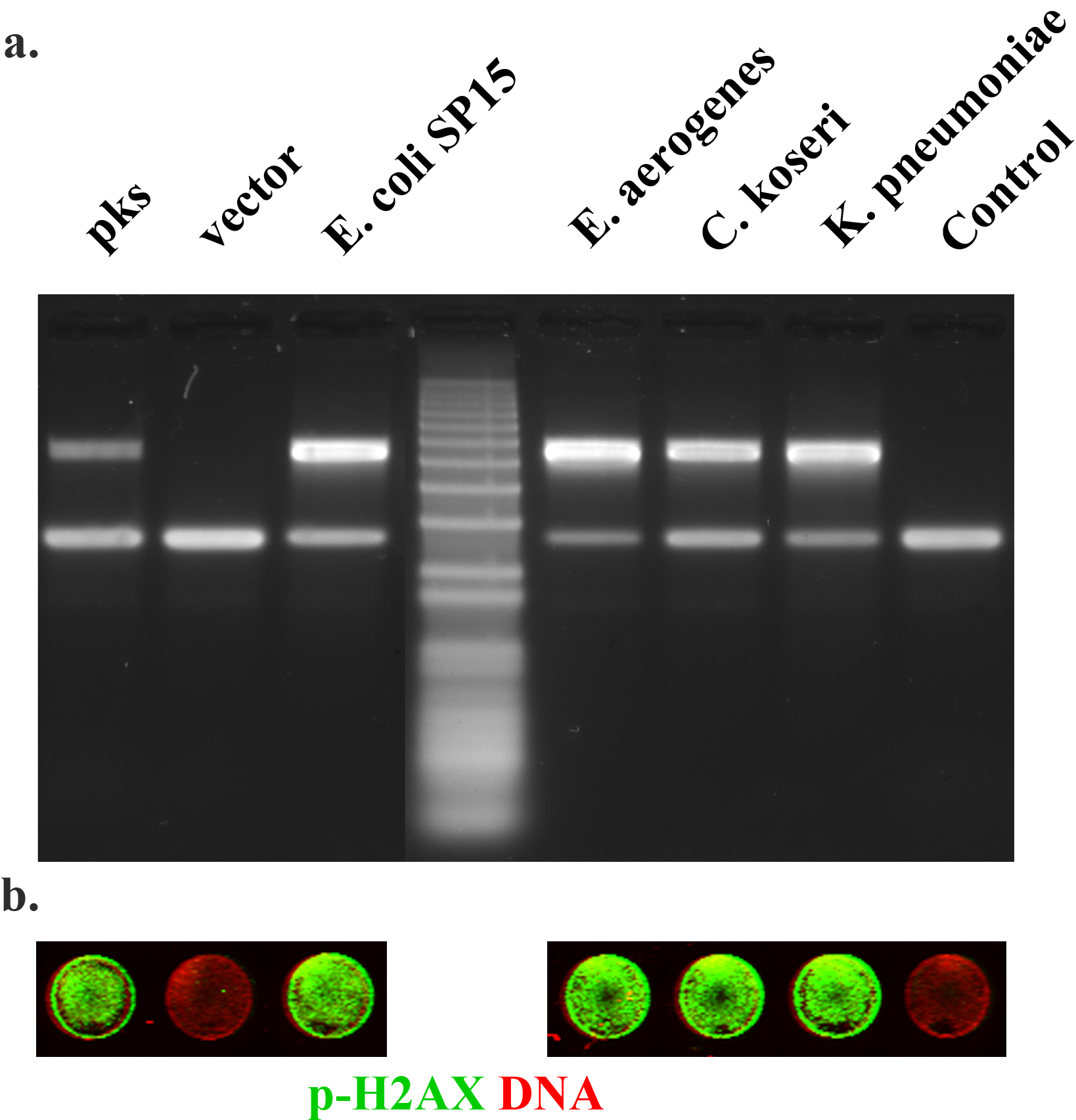

Supplement: FIG S3 [file mbo002183800sf3.tif]

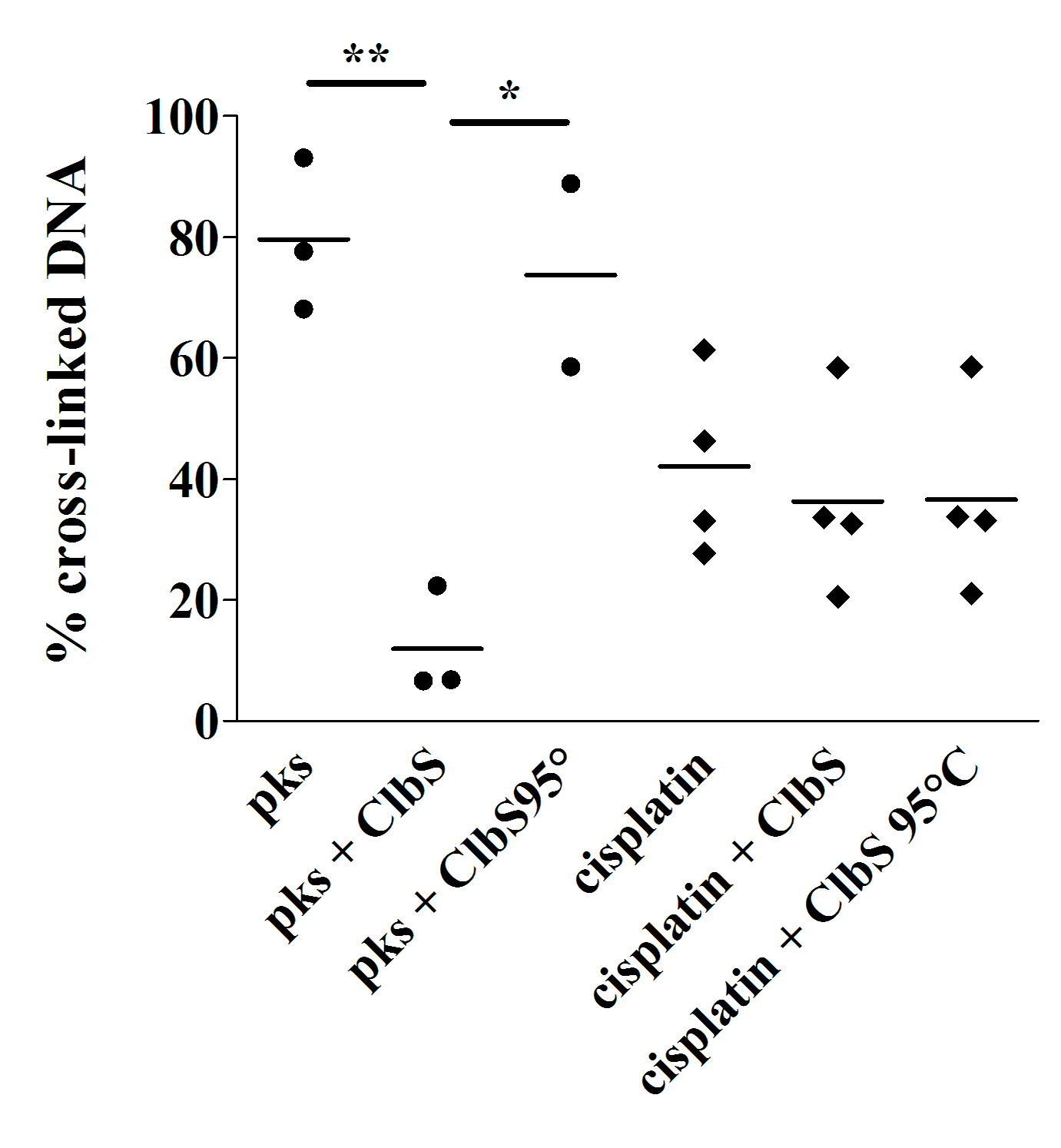

Supplement: FIG S4 [file mbo002183800sf4.tif]

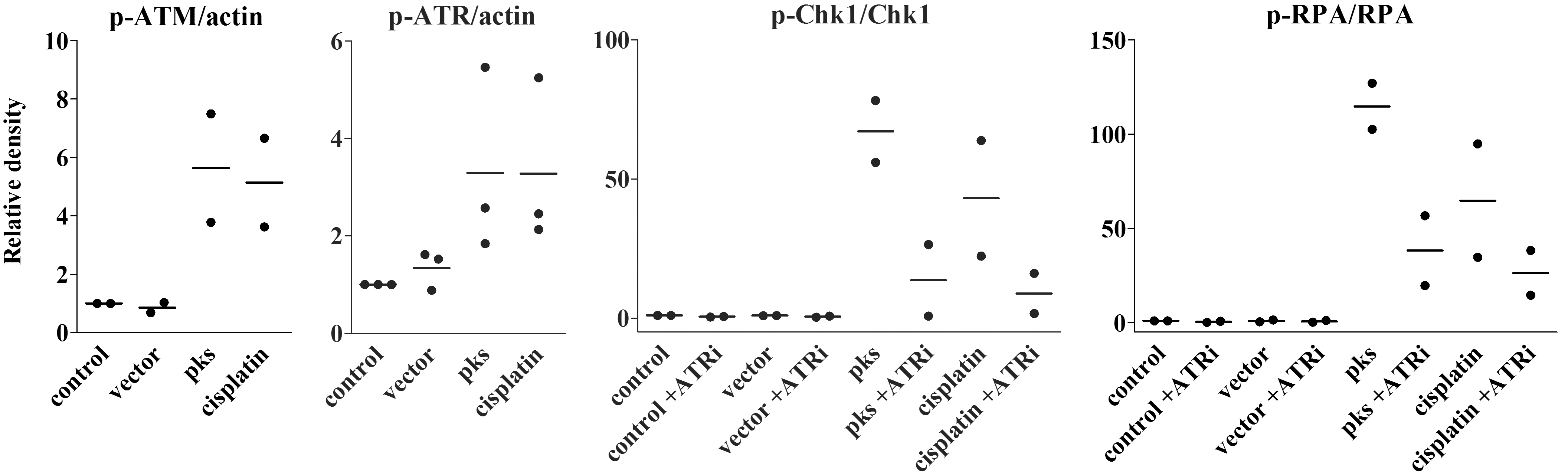

Supplement: FIG S5 [file mbo002183800sf5.tif]

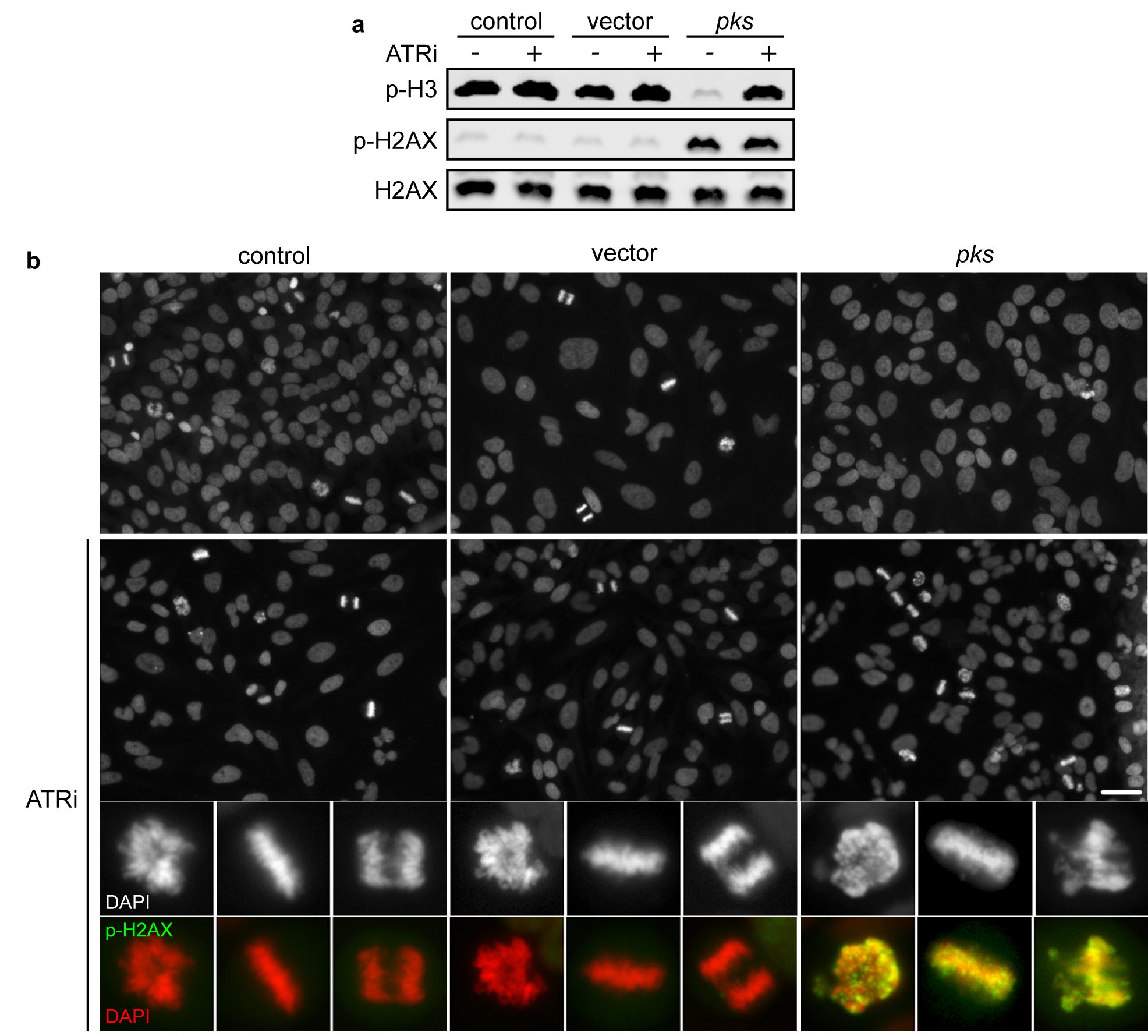

Supplement: FIG S6 [file mbo002183800sf6.tif]

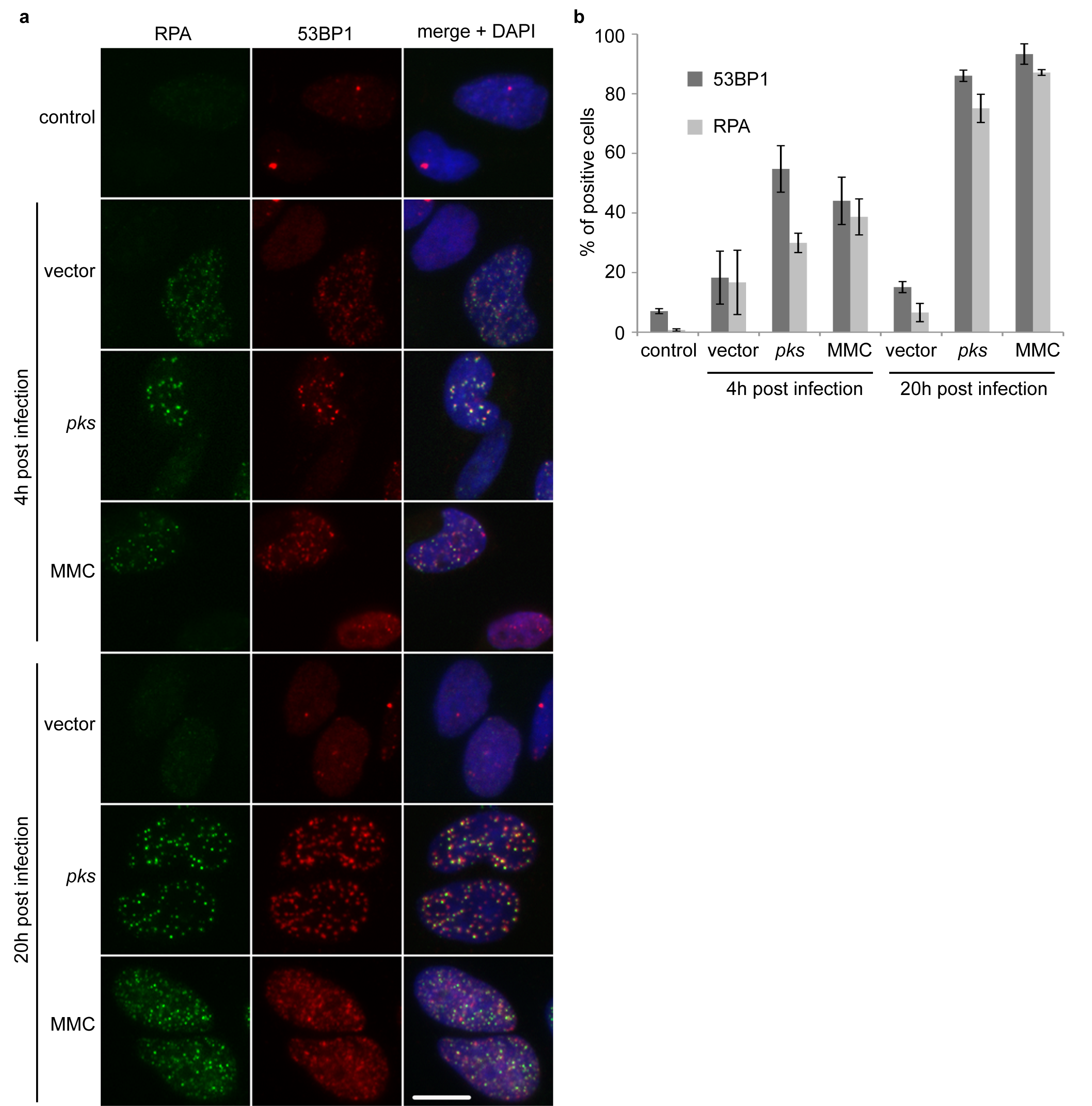

Supplement: FIG S8 [file mbo002183800sf8.tif]

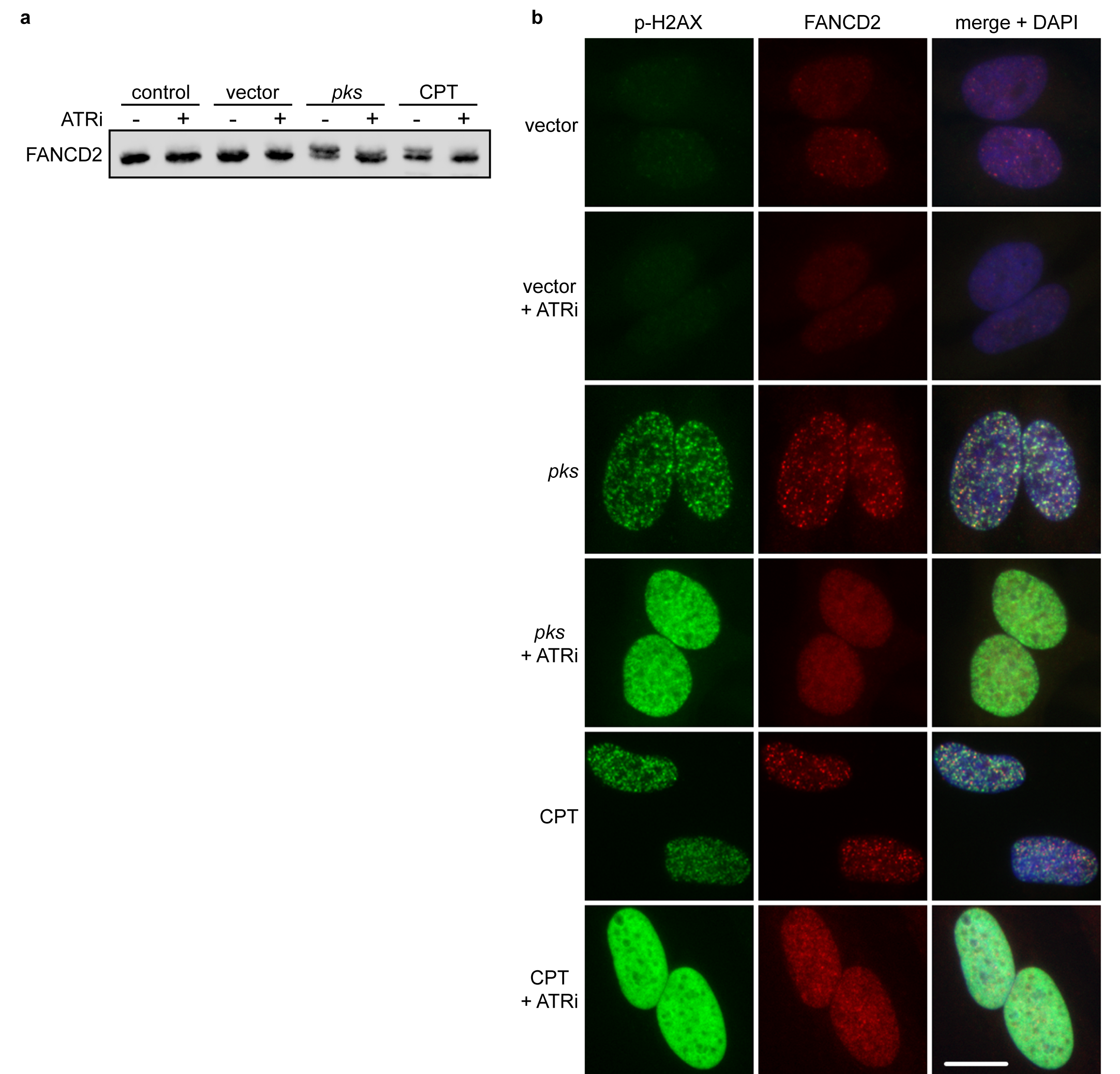

Supplement: FIG S9 [file mbo002183800sf9.tif]

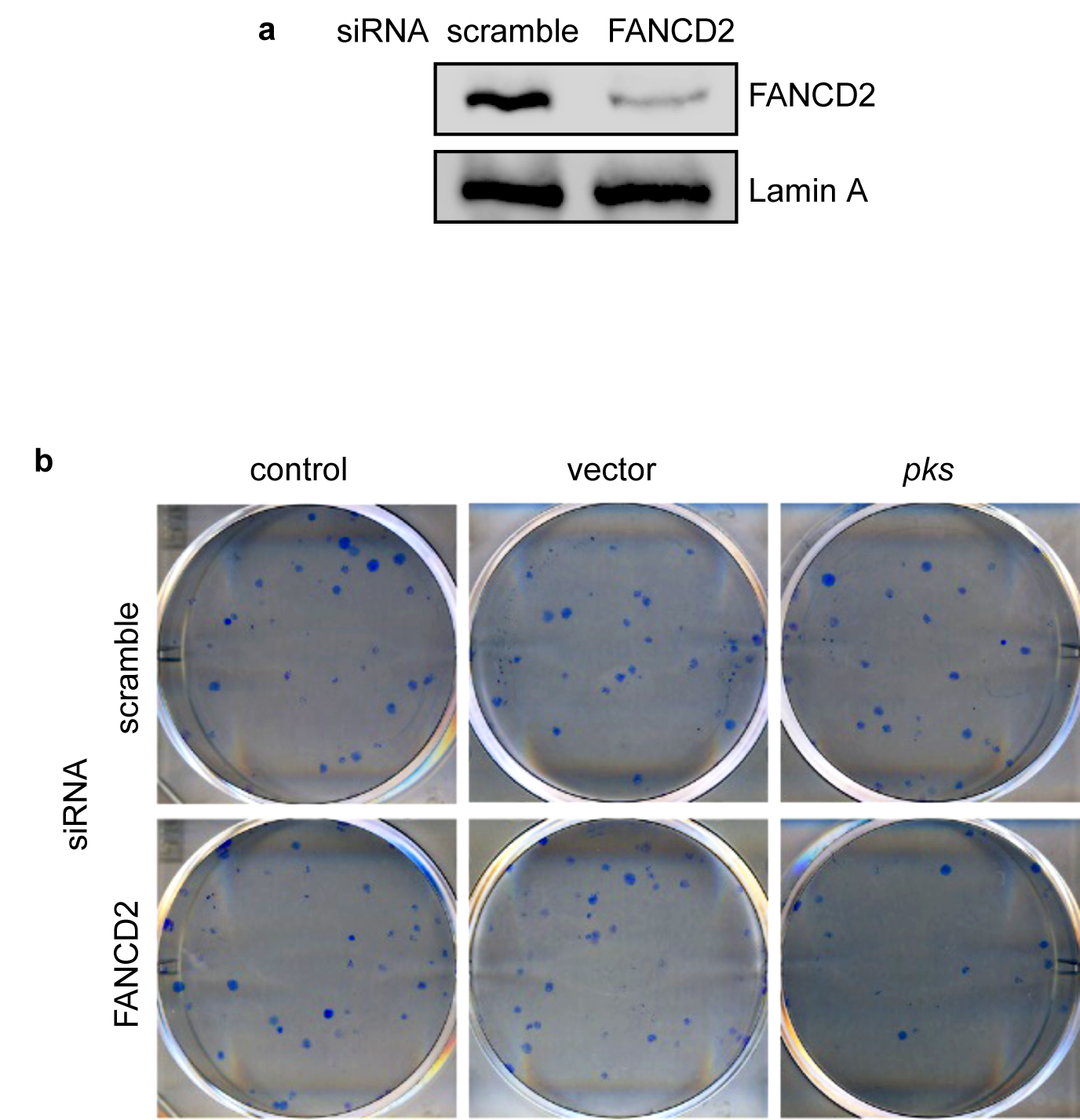

Supplement: FIG S10 [file mbo002183800sf10.tif]
